# Supplementary material for: GIPC proteins negatively modulate Plexind1 signaling during vascular development
Source: eLife. 2019 May 3;8:e30454. doi: 10.7554/eLife.30454 (PMC6499541; doi:10.7554/eLife.30454)
Supplement: Supplementary file 1. — Vectors for expressing PLXND1 and GIPC proteins/fragments, primers for genotyping Tg(fli1a:GAL4FF)ubs4 zebrafish, oligos for assembling DNA templates for in vitro transcription of gRNAs for zebrafish genome editing and for making lentiCRISPRv2-Blast vectors for Cas9 and gRNA coexpression for use in HUVEC, cognate sequences of WT alleles and mutant alleles generated in this study via genome editing, and primers for genotyping mutant alleles generated in this study via genome editing. Related to Figures 1–7, Figure 2—figure supplement 1, Figure 2—figure supplement 2, Figure 4—figure supplement 1, Figure 4—figure supplement 2, Figure 5—figure supplement 1, Figure 7—figure supplement 1 and, Figure 7—figure supplement 2. [file elife-30454-supp1.docx]

**SUPPLEMENTARY FILE 1**

**Vectors for expressing PLXND1 and GIPC proteins/fragments.** Use key: mammalian cells (MC), zebrafish (Z). Vector info key (backbone): **A** (*pcDNA3.1/nV5-DEST-V5;* ThermoFisher Scientific #12290010), **B** (*pFLAG-CMV1*; Sigma Aldrich #E7273), **C** (GAL4-responsive, Gateway and IRES-based bicistronic vector for *Tol2* transgenesis), **D** (GAL4-responsive, Gateway-based vector for *Tol2* transgenesis). Related to **Figure 1** and **Figure 2-figure supplement 2**.

| **Plasmid ID#** | **For expressing these payload(s)** | | **Use** | **Vector**  **info** | **Comments** |
| --- | --- | --- | --- | --- | --- |
|  | **#1** | **#2** |  |  |  |
| 862 | V5-C-mPLXND1^WT^ | n/a | MC | A | N-terminally tagged (V5) cytosolic tail of wild-type murine PLXND1 (aa 1309-1925). |
| 863 | V5-C-mPLXND1Δ^CYSEA^ | n/a | MC | A | N-terminally tagged (V5) cytosolic tail of murine PLXND1 without the C-terminal five aa (CYSEA; aa 1309-1920). |
| 1774 | V5-C-mPLXND1Δ^GBM^ | n/a | MC | A | N-terminally tagged (V5) cytosolic tail of murine PLXND1 without the GBM, the C-terminal nine aa (NIYECYSEA; aa 1309-1916). |
| 864 | FLAG-mGIPC1^WT^ | n/a | MC | B | Murine GIPC1 (333 aa) with N-terminal FLAG tag (8 aa) and linker (2 aa). Gift of Moses Chao (see Acknowledgements). |
| 868 | FLAG-mGIPC1^GH1^ | n/a | MC | B | As FLAG-mGIPC1^WT^ but only the N-terminal 124 aa of mGIPC1 (the region N-terminal to the GH1 domain and the GH1 domain itself). |
| 866 | FLAG-mGIPC1^PDZ^ | n/a | MC | B | As FLAG-mGIPC1^WT^ but only central 110 aa region of mGIPC1 containing the PDZ domain plus 8 N-terminal and 11 C-terminal aa. |
| 1414 | 2xHA-Plxnd1^WT^ | EGFP | Z | C | **Payload #1:** Zebrafish Plxnd1 with N-terminal 2xHA epitope (between the signal peptide and Sema domain). **Payload #2:** cytosolic Enhanced Green Fluorescent Protein. |
| 1685 | 2xHA-Plxnd1Δ^GBM^ | EGFP | Z | C | **Payload #1:** As above, but without the C-terminal nine aa (NIYECSSEA) or GBM. **Payload #2:** cytosolic Enhanced Green Fluorescent Protein. |
| 1828 | EGFP | n/a | Z | D | Cytosolic Enhanced Green Fluorescent Protein. |

**Primers for genotyping *Tg(fli1a:GAL4FF)^ubs4^* zebrafish.** Related to **Figure 2-figure supplement 2**.

| **Primer ID#** | **Oligo name** | **Sequence (5’-to-3’)** |
| --- | --- | --- |
| 11730 | fli1aGAL4-F1 | ACAAAAGACAGGAATGCCC |
| 11731 | fli1aGAL4-R1 | AAATCCAAAATCTCCACCCC |

**Oligos for assembling DNA templates for in vitro transcription of gRNAs for zebrafish genome editing and for making lentiCRISPRv2-Blast vectors for Cas9 and gRNA coexpression for use in HUVEC.** Sequence description of oligos for zebrafish-related use. SP6 promoter (black lowercase italics), the region complementary to the genomic target without the protospacer adjacent motif (red uppercase), the region complementary to the bases between the gene-specific and universal (U) gRNA oligos (blue lowercase). Sequence description of oligos for HUVEC-related use. Region complementary to the genomic target without the protospacer adjacent motif (red upper case), overhangs for cloning into the lentiCRISPRv2-Blast vector (bold black lowercase). The sequences of the non-targeting gRNAs used in HUVEC cells were taken from^102^. The CRISPRscan web tool was used to select *PLXND1* gRNAs. **Related to Figures 2-7, Figure 2-figure supplement 1, Figure 4-figure supplements 1-2, Figure 5-figure supplement 1, Figure 6** and **Figure 7-figure supplements 1-2**.

| **Oligo** | **Oligo sequence (5’-to-3’)** | **Yields gRNA** | **Use** |
| --- | --- | --- | --- |
| gipc1-oligo 1 | *atttaggtgacactata*GGCTCCTCGTTCTCCACCAGgttttagagctagaaatagcaag | gipc1-gRNA1 | Zebrafish |
| gipc2-oligo 1 | *atttaggtgacactata*GGGAAGTCCGCGGTGAACGGgttttagagctagaaatagcaag | gipc2-gRNA1 | Zebrafish |
| gipc2-oligo 2 | *atttaggtgacactata*GGCCGCAGGCTGGCAGGTGGgttttagagctagaaatagcaag | gipc2-gRNA2 | Zebrafish |
| gipc2-oligo 3 | *atttaggtgacactata*GGCACGGGAGTCCCACAGGGgttttagagctagaaatagcaag | gipc2-gRNA3 | Zebrafish |
| gipc2-oligo 4 | *atttaggtgacactata*GTGGAAAACGAGGAGATCGGgttttagagctagaaatagcaag | gipc2-gRNA4 | Zebrafish |
| gipc3-oligo 1 | *atttaggtgacactata*GGGCAGGGCGCGGCGTCCGGgttttagagctagaaatagcaag | gipc3-gRNA1 | Zebrafish |
| gipc3-oligo 2 | *atttaggtgacactata*GGCGCGGCGTCCGGCGGCAGgttttagagctagaaatagcaag | gipc3-gRNA2 | Zebrafish |
| gipc3-oligo 3 | *atttaggtgacactata*GATGGACGCTCAGATGCAGCgttttagagctagaaatagcaag | gipc3-gRNA3 | Zebrafish |
| plxnD1-oligo1 | *atttaggtgacactata*GGTCTATGAGTGCAGCAGCGgttttagagctagaaatagcaag | plxnD1-gRNA1 | Zebrafish |
| U gRNA oligo | **aaaagcaccgactcggtgccactttttcaagttgataacggactagccttattttaa**cttgctatttctagctctaaaac |  | Zebrafish |
| Non-targeting gRNA1-oligo 1 | **caccg**GTAGCGAACGTGTCCGGCGT | Non-targeting gRNA1 | HUVEC |
| Non-targeting  gRNA1-oligo 2 | **aaac**ACGCCGGACACGTTCGCTAC**c** |  | HUVEC |
| Non-targeting gRNA2-oligo 1 | **caccg**GACCGGAACGATCTCGCGTA | Non-targeting gRNA2 | HUVEC |
| Non-targeting gRNA2-oligo 2 | **aaac**TACGCGAGATCGTTCCGGTC**c** |  | HUVEC |
| PLXND1-KO1  oligo 1 | **caccg**TGGACCCGCACACGACTACC | PLXND1-KO1 | HUVEC |
| PLXND1-KO1  oligo 2 | **aaac**GGTAGTCGTGTGCGGGTCCA**c** |  | HUVEC |
| PLXND1-KO2  oligo 1 | **caccg**CCACGGTGGACGCGTTCGGG | PLXND1-KO2 | HUVEC |
| PLXND1-KO2  oligo 2 | **aaac**CCCGAACGCGTCCACCGTGGc |  | HUVEC |

**Cognate sequences of WT alleles and mutant alleles generated in this study via genome editing.** The differences in length between the wild-type (WT) and mutant (M) alleles are indicated for the latter. In the WT allele, the target genomic sequence complementary to the gRNA is highlighted in yellow. The two bases between which Cas9 was predicted to cut are bolded. The PAM (Protospacer Adjacent Motif) is highlighted in blue. Related to **Figures 2-7, Figure 2-figure supplement 1, Figure 4-figure supplements 1-2, Figure 5-figure supplement 1, Figure 6** and **Figure 7-figure supplements 1-2**.

| **Gene** | **Mutagenic gRNA (s)** | **Allele** | **gDNA sequence (5’ to 3’)** | **Use** |
| --- | --- | --- | --- | --- |
| ***gipc1*** | N/A | **WT** | atgccacttggattgggacgcagaaagaaggcgtctccgct**gg**tggagaacgaggaggctgaacccatccgggct | Zebrafish |
|  | gipc1-gRNA1 | ***gipc1^skt1^*** (M -4) | atgccacttggattgggacgcagaaagaaggcgtctctttcgagaacgaggaggctgaacccatccgggct | Zebrafish |
|  | N/A | **WT** | atgccacttggattgggacgcagaaagaaggcgtctccgct**gg**tggagaacgaggaggctgaacccatccgggct | Zebrafish |
|  | gipc1-gRNA1 | ***gipc1^skt2^*** (M +7) | atgccacttggattgggacgcagaaagaaggcgtctccgctggagttcgctggagaacgaggaggctgaacccatccgggct | Zebrafish |
| ***gipc2*** | N/A | **WT** | gaacttagtggaaaacgaggaga**tc**ggcggacatgcagttgtcgggaagtccgcggtga**ac**ggcggaggactccctcctccacctgccagcctgc | Zebrafish |
|  | gipc2-gRNA1-4 | ***gipc2^skt4^*** (M -2) | gaacttagtggaaaacgaggagacatgcaggcggacatgcagtcggcggacatgcagttgtcgggaagtccgcggtgaa  gtcctccagcctgc | Zebrafish |
|  | N/A | **WT** | ggaaaacgaggagatcggcggacatgcagttgtcgggaagtccgcggtga**ac**ggcggaggactccctcctcc**ac**ctg ccagcctgcggccgaaactggtgttccatacgcagctcgcgcac | Zebrafish |
|  | gipc2-gRNA1-4 | ***gipc2^skt3^*** (M -46) | ggaaaacgaggagatcggcggaggactcctcccagcctgcggccgaaactggtgttccatacgcagctcgcgcac | Zebrafish |
| ***gipc3*** | N/A | **WT** | gtgatggacgctcagatg**ca**gcaggactcccagaacctccagcccatgcagaacggagaggccatgagccccggcccgcaggactccacgggtcccccaggggacgaggagagccagagcactgtcccgtccgccccgccgct**gc**cgcc**gg**acgccgcgccctgccccaggcccaaactggtgttt | Zebrafish |
|  | gipc3-gRNA1-3 | ***gipc3^skt5^*** (M -38) | gtgatggacgctcagacgctcagcaggtctcccagaacctccagcccatgcagaacggagaggccatgagccccggcccgcaggactccacgggtcccccaggggacgaggagagccagagcactgtcccaggcccaaactggtgttt | Zebrafish |
| ***plxnd1*** | N/A | **WT** | tggtggaggacaacatctatgagtgcagc**ag**cgaggcctgaacacacacacacacacacacacgcacacacacactcctgaatgcctgraaactgcctgcttaaagggccagttccccctgaaatga | Zebrafish |
|  | plxnd1-gRNA1 | ***plxnd1^skt6^*** (M -4) | tggtggaggacaacatctatgagtctgcgaggcctgaacacacacacacacacacacacacacacactcctgaatgcctggaaacagcctgcttaaagggccagttccccctgaaatga | Zebrafish |
| ***PLXND1*** | N/A | **WT** | ccccggccagggcctggt**ag**tcgtgtgcgggtccatctaccagggcttctgccagctgcggcgccggggcaacatctcggccgtg | HUVEC |
|  | PLXND1-gRNA1 | ***PLXND1^1KO#1^*** (M -4) | ccccggccagggccggtcgtgtgcgggtccatctaccagggcttctgccagctgcggcgccggggcaacatctcggccgtg | HUVEC |
|  |  | ***PLXND1^2KO#1^*** (M -1) | ccccggccagggccagctgtcgtgtgcgggtccatctaccagggcttctgccagctgcggcgccggggcaacatctcggccgtg | HUVEC |
|  | N/A | **WT** | ctgaacgtggcggccaaccacc**cg**aacgcgtccaccgtggggctagttctgcctcccgccgcgggcgcg | HUVEC |
|  | PLXND1-gRNA2 | ***PLXND1^1KO#2^*** (M +1) | ctgaacgtggcggccaaccacccagaacgcgtccaccgtggggctagttctgcctcccgccgcgggcgcggggggcagccgcctgctcgtgggcgccacgtacaccggttacggcagctccttcttcc | HUVEC |
|  |  | ***PLXND1^2KO#2^*** (M -14) | ctgaacgtggaacgcgtccaccgtggggctagttctgcctcccgccgcgggcgcggggggcagccgcctgctcgtgggcgccacgtacaccggttacggcagctccttcttcc | HUVEC |

**Primers for genotyping mutant alleles generated in this study via genome editing.** Related to **Figures 2-7, Figure 2-figure supplement 1, Figure 4-figure supplements 1-2, Figure 5-figure supplement 1, Figure 6** and **Figure 7-figure supplements 1-2**.

| **Mutant alleles** | **Primer ID#** | **Oligo name** | **Sequence (5’-to-3’)** | **Use** |
| --- | --- | --- | --- | --- |
| *gipc1^skt1^* and *gipc1^skt2^* | 12891 | gipc1-F1 | ATTTGGGAACATAAAGAACG | Zebrafish |
|  | 12889 | gipc1-R1 | CTCTGTTGGAGGAATCCC | Zebrafish |
| *gipc1^skt1^* and *gipc1^skt2^* | 13655 | gipc1-F2 | ATGCCACTTGGATTGGGACGC | Zebrafish |
|  | 13653 | gipc1-R2 | CTCTGTTGGAGGAATCCCAAAGGC | Zebrafish |
| *gipc2^skt3^* and *gipc2^skt4^* | 13667 | gipc2-F1 | GCTCGAACTTAGTGGAAAACG | Zebrafish |
|  | 13422 | gipc2-R1 | TGGACTTAAATTAAAGGCTTCTGC | Zebrafish |
|  | 13421 | gipc2-F2 | CCGCATACAGTCAATTTATACTCG | Zebrafish |
| *gipc3^skt5^* | 13510 | gipc3-F1 | AGGTGTCGTCTGCTCTCCAGTGTC | Zebrafish |
|  | 13512 | gipc3-R1 | TGCTATTTTGGCGTAGAGCTCCC | Zebrafish |
| *plxnd1^skt6^* | 11844 | plxnD1-F1 | TACGCCAAGAGATACCGTG | Zebrafish |
|  | 11845 | plxnD1-R1 | AATGCTGGAAGCCTGTAAC | Zebrafish |
|  | 12532 | plxnD1-F2 | TGCGCTGGACTCGAACCC | Zebrafish |
| *plxnd1^fov01b^* | 13498 | plxnD1-F3 | CCCGGAGATCGCCATCCG | Zebrafish |
|  | 10167 | plxnD1-R2 | CCCTGGATGACGCTGTCCA | Zebrafish |
|  | 13646 | plxnD1-F4 | CTTCACCTACGACATCAACC | Zebrafish |
|  | 13647 | plxnD1-R3 | TCTCCTCCACCTCGGCCACG | Zebrafish |
| *PLXND1^1KO#1^*, *PLXND1^2KO#1^*, *PLXND1^1KO#2^* and, *PLXND1^2KO#2^* | 13744 | PLXND1-F1 | CAACCGCCTCTATCAGCTGT | HUVEC |
|  | 13863 | PLXND1-R1 | GGAGGGGTTGAGGTCGAAGGTG | HUVEC |
