# Supplementary material for: GIPC proteins negatively modulate Plexind1 signaling during vascular development
Source: eLife. 2019 May 3;8:e30454. doi: 10.7554/eLife.30454 (PMC6499541; doi:10.7554/eLife.30454)
Supplement: Supplementary file 2. — Related to Figure 1. [file elife-30454-supp2.docx]

**SUPPLEMENTARY FILE 2**

**Raw densitometry values of tagged proteins in Western Blots of co-IP experiments.** Related to **Figure 1**.

|  | **Independent experiment** | **Co-transfection** | | |
| --- | --- | --- | --- | --- |
|  |  | **FLAG-mGIPC1**  **+**  **V5-C-mPLXND1^WT^** | **FLAG-mGIPC1**  **+**  **V5-C-mPLXND1Δ^CYSEA^** | **FLAG-mGIPC1**  **+**  **V5-C-mPLXND1Δ^GBM^** |
| **V5^Co-IP^** | **1** | 40.49 | 11.71 | 6.95 |
|  | **2** | 30.40 | 15.81 | 4.81 |
|  | **3** | 35.12 | 10.80 | 7.73 |
| **V5^TCL^** | **1** | 111.14 | 99.71 | 96.72 |
|  | **2** | 88.68 | 74.16 | 72.02 |
|  | **3** | 93.11 | 79.92 | 74.83 |
| **FLAG^IP^** | **1** | 99.95 | 92.75 | 92.15 |
|  | **2** | 79.33 | 67.61 | 69.33 |
|  | **3** | 105.62 | 90.89 | 82.04 |
| **FLAG^TCL^** | **1** | 82.23 | 90.09 | 84.87 |
|  | **2** | 53.03 | 57.97 | 51.36 |
|  | **3** | 69.09 | 55.44 | 53.09 |

**Average densitometry values of tagged proteins in Western Blots of co-IP experiments and their ratios.** The Relative binding V5/FLAG and Relative abundance V5^TCL^/FLAG^TCL^ values were calculated using the values of the FLAG-mGIPC1 + V5-C-mPLXND1^WT^ co-transfection as the reference. Related to **Figure 1**.

|  | | **Co-transfection** | | |
| --- | --- | --- | --- | --- |
|  |  | **FLAG-mGIPC1**  **+**  **V5-C-mPLXND1^WT^** | **FLAG-mGIPC1**  **+**  **V5-C-mPLXND1Δ^CYSEA^** | **FLAG-mGIPC1**  **+**  **V5-C-mPLXND1Δ^GBM^** |
| **Average**  **densitometry** | **V5^Co-IP^** | 35.34 | 12.77 | 6.50 |
|  | **V5^TCL^** | 97.64 | 84.60 | 81.19 |
|  | **FLAG^IP^** | 94.97 | 83.75 | 81.18 |
|  | **FLAG^TCL^** | 68.12 | 67.83 | 63.11 |
| **V5** | **V5^Co-IP^/V5^TCL^** | 0.36 | 0.15 | 0.08 |
| **FLAG** | **FLAG^IP^/FLAG^TCL^** | 1.39 | 1.23 | 1.29 |
| **Relative binding**  **V5/FLAG** | Ratio | 0.26 | 0.12 | 0.06 |
|  | **Percentual**  **mean ± S.E.M.** | **100 ± 8.20** | **48.80 ± 11.45** | **23.60 ± 2.20** |
| **Relative abundance**  **V5^TCL^/FLAG^TCL^** | Ratio | 1.46 | 1.27 | 1.32 |
|  | **Percentage** | **100 %** | **87.56 %** | **90.40 %** |

**Statistical significances**. A one-way ANOVA, followed by a Tukey post hoc analysis was conducted to determine if the percentual V5/FLAG relative binding in Western Blots of co-IP experiments of FLAG-mGIPC1 and V5-C-mPLXND1 forms was significantly different (p < .05) between the three tested pairs of these proteins (n = 3 independent experiments for each protein pair). Combinations of protein pairs with significantly different percentual V5/FLAG relative binding are highlighted in green. Related to **Figure 1C**.

|  | **FLAG-mGIPC1**  **+ V5-C-mPLXND1Δ^CYSEA^** | **FLAG-mGIPC1**  **+ V5-C-mPLXND1Δ^GBM^** |
| --- | --- | --- |
| **FLAG-mGIPC1**  **+**  **V5-C-mPLXND1^WT^** | .011 | .001 |
| **FLAG-mGIPC1**  **+ V5-C-mPLXND1Δ^CYSEA^** |  | .154 |
