# Supplementary material for: GIPC proteins negatively modulate Plexind1 signaling during vascular development
Source: eLife. 2019 May 3;8:e30454. doi: 10.7554/eLife.30454 (PMC6499541; doi:10.7554/eLife.30454)
Supplement: Supplementary file 3. [file elife-30454-supp3.docx]

**SUPPLEMENTARY FILE 3**

***plxnd1^skt6^* complementation of *plxnd1^fov01b^***

**Quantification. Percentage of Se-DLAV truncations in 32 hpf embryos of the indicated genotypes** belonging to each of the following four phenotypic classes. Truncated: maximal, moderate and, minimal. Non-truncated: Full. All the *plxnd1^fov01b^* mutants (12 embryos) displayed hyperangiogenic vascular mispatterning (not tabulated). Related to **Figure 2C-E**.

| **Genotype** | **Scored Se-DLAV** | | | | | | **Total embryos**  **analyzed** | **Se-DLAV/**  **embryo** |
| --- | --- | --- | --- | --- | --- | --- | --- | --- |
|  | **Truncated** | | | | **Non-truncated** | **Total**  **scored** |  |  |
|  | **Maximal** | **Moderate** | **Minimal** | **Total** | **Full** |  |  |  |
| **WT** | 0 | 0 | 0 | 0 | 124 | 124 | 11 | 11.27 |
|  | 0 % | 0 % | 0 % | **0** % | **100** % |  |  |  |
| ***plxnd1^fov01b^/plxnd1^skt6^***  transheterozygotes | 0 | 0 | 0 | 0 | 162 | 162 | 16 | 10.13 |
|  | 0 % | 0 % | 0 % | **0** % | **100** % |  |  |  |

**Quantification. Penetrance of Se-DLAV truncations in 32 hpf embryos of the indicated genotypes.** All the *plxnd1^fov01b^* mutants (12 embryos) displayed hyperangiogenic vascular mispatterning (not tabulated). Related to **Figure 2C-E**.

| **Genotype** | **Embryos with**  **Se-DLAV truncations** | **Embryos without**  **Se-DLAV truncations** | **Total embryos analyzed** |
| --- | --- | --- | --- |
| **WT** | 0 | 11 | 11 |
|  | **0 %** | **100 %** | **100 %** |
| ***plxnd1^fov01b^/plxnd1^skt6^***  transheterozygotes | 0 | 16 | 16 |
|  | **0 %** | **100 %** | **100 %** |

**Quantification. Expressivity of Se-DLAV truncations in 32 hpf embryos of the indicated genotypes.** All the *plxnd1^fov01b^* mutants (12 embryos) displayed hyperangiogenic vascular mispatterning (not tabulated). Related to **Figure 2C-E**.

| **Genotype** | **Se-DLAV in embryos with Se-DLAV truncations** | | | | | | **Embryos with Se-DLAV truncations** |
| --- | --- | --- | --- | --- | --- | --- | --- |
|  | **Truncated** | | | | **Non-truncated** | **Total**  **Se-DLAV**  **scored** |  |
|  | **Maximal** | **Moderate** | **Minimal** | **Total** | **Full** |  |  |
| **WT** | 0 | 0 | 0 | 0 | 0 | 0 | 0/11 |
|  | 0 % | 0 % | 0 % | **0 %** | **0 %** | 0 % |  |
| ***plxnd1^fov01b^/plxnd1^skt6^***  transheterozygotes | 0 | 0 | 0 | 0 | 0 | 0 | 0/16 |
|  | 0 % | 0 % | 0 % | **0 %** | **0 %** | 0 % |  |

**Comparison of the vascular phenotypes of homozygous WT and homozygous *plxnd1^skt6^* mutant siblings**

**Quantification. Percentage of Se-DLAV truncations in 32 hpf embryos of the indicated genotypes** belonging to each of the following four phenotypic classes. Truncated: maximal, moderate and, minimal. Non-truncated: Full. Related to **Figure 2F-I**.

| **Genotype** | **Scored Se-DLAV** | | | | | | **Total embryos**  **analyzed** | **Se-DLAV/**  **embryo** |
| --- | --- | --- | --- | --- | --- | --- | --- | --- |
|  | **Truncated** | | | | **Non-truncated** | **Total**  **scored** |  |  |
|  | **Maximal** | **Moderate** | **Minimal** | **Total** | **Full** |  |  |  |
| **WT**  **(siblings)** | 0 | 0 | 0 | 0 | 126 | 126 | 12 | 10.50 |
|  | 0 % | 0 % | 0 % | **0 %** | **100 %** |  |  |  |
| ***plxnd1^skt6^***  **(siblings)** | 2 | 2 | 0 | 4 | 120 | 124 | 12 | 10.33 |
|  | 1.6 % | 1.6 % | 0 % | **3.2 %** | **96.8** % |  |  |  |

**Quantification. Penetrance of Se-DLAV truncations in 32 hpf embryos of the indicated genotypes.** Related to **Figure 2-figure supplement 1A**.

| **Genotype** | **Embryos with**  **Se-DLAV truncations** | **Embryos without**  **Se-DLAV truncations** | **Total embryos analyzed** |
| --- | --- | --- | --- |
| **WT**  **(siblings)** | 0 | 12 | 12 |
|  | **0 %** | **100 %** | **100 %** |
| ***plxnd1^skt6^***  **(siblings)** | 3 | 9 | 12 |
|  | **25 %** | **75 %** | **100 %** |

**Quantification. Expressivity of Se-DLAV truncations in 32 hpf embryos of the indicated genotypes.** Related to **Figure 2-figure supplement 1B**.

| **Genotype** | **Se-DLAV in embryos with Se-DLAV truncations** | | | | | | **Embryos with Se-DLAV truncations** |
| --- | --- | --- | --- | --- | --- | --- | --- |
|  | **Truncated** | | | | **Non-truncated** | **Total**  **Se-DLAV**  **scored** |  |
|  | **Maximal** | **Moderate** | **Minimal** | **Total** | **Full** |  |  |
| **WT**  **(siblings)** | 0 | 0 | 0 | 0 | 0 | 0 | 0/12 |
|  | 0 % | 0 % | 0 % | **0** % | **0** % | 0 % |  |
| ***plxnd1^skt6^***  **(siblings)** | 2 | 2 | 0 | 4 | 28 | 32 | 3/12 |
|  | 6.25 % | 6.25 % | 0 % | **12.5** % | **87.5** % | 100 % |  |

**Significance values (*p*) obtained by comparing the distributions of Se-DLAV truncations between WT and *plxnd1^skt6^* mutants at 32 hpf.** Genotypes are shown in bold text with gray highlights. Distributions involve the following four phenotypic classes. Truncated: maximal, moderate and, minimal. Non-truncated: Full. Significance values were calculated using two-sided Fisher’s Exact tests, *p* < 0.05. No significant differences were found. See **Figure 2I**.

| **Genotype pair** | | **Comparison of the distributions of Se-DLAV truncations** | | | | |
| --- | --- | --- | --- | --- | --- | --- |
|  |  | **All four categories** | **Truncated**  ***vs.***  **not-truncated** | **Maximal**  ***vs.***  **the other three categories** | **Moderate**  ***vs.***  **the other three categories** | **Minimal**  ***vs.***  **the other three categories** |
| **WT (siblings)** | ***plxnd1^skt6^***  **(siblings)** | .05905 | .05905 | .24501 | .24501 | 1 |

**Significance value (*p*) obtained by comparing the penetrance of Se-DLAV truncations (embryos with Se-DLAV truncations *vs.* embryos without Se-DLAV truncations) between WT and *plxnd1^skt6^* mutants at 32 hpf.** Genotypes are shown in bold text with gray highlights. The significance value was calculated using a two-sided Fisher’s Exact test, *p* < 0.05. No significant difference was found. See **Figure 2-figure supplement 1A**.

| **Genotype pair** | | **Significance value (*p*)** |
| --- | --- | --- |
| **WT**  **(siblings)** | ***plxnd1^skt6^***  **(siblings)** | .21739 |

**Mosaic transgenic endothelial expression of tagged forms of zebrafish Plxnd1 in *plxnd1^fov01b^* null mutants**

**Quantification of the phenotype of Se and DLAV clones with exogenous expression of the indicated 2xHA-Plxnd1 forms.** Related to **Figure 2-figure supplement 2J**.

| **Exogenous 2xHA-Plxnd1** | | **Phenotype** | | **Total** | **Embryos** |
| --- | --- | --- | --- | --- | --- |
|  |  | **WT-like** | ***plxnd1* null-like** |  |  |
| **Plxnd1^WT^** | **Clones** | 19 | 2 | 21 | 15 |
|  | **%** | **90.48 %** | **9.52 %** | **100 %** |  |
| **Plxnd1Δ^GBM^** | **Clones** | 8 | 1 | 9 | 17 |
|  | **%** | **88.89 %** | **11.11 %** | **100 %** |  |
