# Supplementary material for: GIPC proteins negatively modulate Plexind1 signaling during vascular development
Source: eLife. 2019 May 3;8:e30454. doi: 10.7554/eLife.30454 (PMC6499541; doi:10.7554/eLife.30454)
Supplement: Supplementary file 4. [file elife-30454-supp4.docx]

**SUPPLEMENTARY FILE 4**

**Quantification. Percentage of Se-DLAV truncations in 32 hpf embryos** **of the indicated genotype and treatment combinations** belonging to each of the following four phenotypic classes. Truncated: maximal, moderate and, minimal. Non-truncated: Full. Related to **Figure 3E.**

| **Genotype and treatment** | **Scored Se-DLAV** | | | | | | **Total embryos**  **analyzed** | **Se-DLAV/**  **embryo** |
| --- | --- | --- | --- | --- | --- | --- | --- | --- |
|  | **Truncated** | | | | **Non-truncated** | **Total scored** |  |  |
|  | **Maximal** | **Moderate** | **Minimal** | **Total** | **Full** |  |  |  |
| **Wild-type**  **(DMSO)** | 0 | 7 | 41 | 48 | 264 | 312 | 28 | 11.14 |
|  | 0 % | 2.2 % | 13.1 % | **15.3** % | **84.7** % |  |  |  |
| ***plxnd1^skt6^***  **(DMSO)** | 0 | 10 | 105 | 115 | 169 | 284 | 26 | 10.92 |
|  | 0 % | 3.5 % | 37 % | **40.5** % | **59.5** % |  |  |  |
| **Wild-type**  **(SU5416)** | 11 | 66 | 129 | 206 | 116 | 322 | 29 | 11.10 |
|  | 3.4 % | 20.5 % | 40.1 % | **64 %** | **36 %** |  |  |  |
| ***plxnd1^skt6^***  **(SU5416)** | 64 | 107 | 90 | 261 | 59 | 320 | 28 | 11.43 |
|  | 20 % | 33.4 % | 28.1 % | **81.5 %** | **18.5** % |  |  |  |

**Quantification. Penetrance of Se-DLAV truncations in 32 hpf embryos of the indicated genotype and treatment combinations.** Related to **Figure 3-figure supplement 1A**.

| **Genotype and treatment** | **Embryos with**  **Se-DLAV truncations** | **Embryos without**  **Se-DLAV truncations** | **Total embryos analyzed** |
| --- | --- | --- | --- |
| **Wild-type**  **(DMSO)** | 10 | 18 | 28 |
|  | **35.7 %** | **64.3 %** | **100 %** |
| ***plxnd1^skt6^***  **(DMSO)** | 19 | 7 | 26 |
|  | **73.1 %** | **26.9 %** | **100 %** |
| **Wild-type**  **(SU5416)** | 26 | 3 | 29 |
|  | **89.7 %** | **10.3 %** | **100 %** |
| ***plxnd1^skt6^***  **(SU5416)** | 28 | 0 | 28 |
|  | **100 %** | **0 %** | **100 %** |

**Quantification. Expressivity of Se-DLAV truncations in 32 hpf embryos of the indicated genotype and treatment combinations.** Related to **Figure 3-figure supplement 1B**.

| **Genotype and treatment** | **Se-DLAV in embryos with Se-DLAV truncations** | | | | | | **Embryos with Se-DLAV truncations** |
| --- | --- | --- | --- | --- | --- | --- | --- |
|  | **Truncated** | | | | **Non-truncated** | **Total**  **Se-DLAV**  **scored** |  |
|  | **Maximal** | **Moderate** | **Minimal** | **Total** | **Full** |  |  |
| **Wild-type**  **(DMSO)** | 0 | 7 | 36 | 43 | 69 | 112 | 10/28 |
|  | 0 % | 6.3 % | 32.1 % | **38.4 %** | **61.6 %** | 100 % |  |
| ***plxnd1^skt6^***  **(DMSO)** | 0 | 10 | 99 | 109 | 54 | 163 | 19/26 |
|  | 0 % | 6.1 % | 60.7 % | **66.8 %** | **33.2 %** | 100 % |  |
| **Wild-type**  **(SU5416)** | 11 | 66 | 129 | 206 | 86 | 292 | 26/29 |
|  | 3.8 % | 22.6 % | 44.2 % | **70.6 %** | **29.4 %** | 100 % |  |
| ***plxnd1^skt6^***  **(SU5416)** | 64 | 107 | 90 | 261 | 59 | 320 | 28/28 |
|  | 20 % | 33.4 % | 28.1 % | **81.5** **%** | **18.5** **%** | 100 % |  |

**Significance values (*p*) obtained by comparing the distributions of Se-DLAV truncations between embryos of the indicated genotype and treatment combinations at 32 hpf.** Genotype and treatment combinations are shown in bold text with gray highlights. Distributions involve the following four phenotypic classes. Truncated: maximal, moderate and, minimal. Non-truncated: Full. Significance values were calculated using two-sided Fisher’s Exact tests and significant differences (*p* < .0083; highlighted in green) assigned using a Bonferroni type adjustment for six pairwise genotype comparisons (0.05/6 = .0083). See **Figure 3E**.

**Significance values (*p*) of pairwise comparisons of the distribution of Se-DLAV truncations (all four categories).**

|  | **WT (SU5416)** | ***plxnd1^skt6^* (DMSO)** | ***plxnd1^skt6^* (SU5416)** |
| --- | --- | --- | --- |
| **WT (DMSO)** | < .0001 | < .0001 | < .0001 |
| **WT (SU5416)** |  | < .0001 | < .0001 |
| ***plxnd1^skt6^* (DMSO)** |  |  | < .0001 |
| ***plxnd1^skt6^* (SU5416)** |  |  |  |

**Significance values (*p*) of pairwise comparisons of the distribution of Se-DLAV truncations (truncated *vs.* not-truncated).**

|  | **WT (SU5416)** | ***plxnd1^skt6^* (DMSO)** | ***plxnd1^skt6^* (SU5416)** |
| --- | --- | --- | --- |
| **WT (DMSO)** | < .0001 | < .0001 | < .0001 |
| **WT (SU5416)** |  | < .0001 | < .0001 |
| ***plxnd1^skt6^* (DMSO)** |  |  | < .0001 |
| ***plxnd1^skt6^* (SU5416)** |  |  |  |

**Significance values (*p*) of pairwise comparisons of the distribution of Se-DLAV truncations (maximal *vs.* the other three categories).**

|  | **WT (SU5416)** | ***plxnd1^skt6^* (DMSO)** | ***plxnd1^skt6^* (SU5416)** |
| --- | --- | --- | --- |
| **WT (DMSO)** | .00091 | 1 | < .0001 |
| **WT (SU5416)** |  | .00109 | < .0001 |
| ***plxnd1^skt6^* (DMSO)** |  |  | < .0001 |
| ***plxnd1^skt6^* (SU5416)** |  |  |  |

**Significance values (*p*) of pairwise comparisons of the distribution of Se-DLAV truncations (moderate *vs.* the other three categories).**

|  | **WT (SU5416)** | ***plxnd1^skt6^* (DMSO)** | ***plxnd1^skt6^* (SU5416)** |
| --- | --- | --- | --- |
| **WT (DMSO)** | < .0001 | .46135 | < .0001 |
| **WT (SU5416)** |  | < .0001 | .00026 |
| ***plxnd1^skt6^* (DMSO)** |  |  | < .0001 |
| ***plxnd1^skt6^* (SU5416)** |  |  |  |

**Significance values (*p*) of pairwise comparisons of the distribution of Se-DLAV truncations (minimal *vs.* the other three categories).**

|  | **WT (SU5416)** | ***plxnd1^skt6^* (DMSO)** | ***plxnd1^skt6^* (SU5416)** |
| --- | --- | --- | --- |
| **WT (DMSO)** | < .0001 | < .0001 | < .0001 |
| **WT (SU5416)** |  | 0. 45258 | .00156 |
| ***plxnd1^skt6^* (DMSO)** |  |  | .02337 |
| ***plxnd1^skt6^* (SU5416)** |  |  |  |

**Significance values (*p*) obtained by comparing the penetrance and expressivity of Se-DLAV angiogenesis deficits between embryos of the indicated genotype and treatment combinations at 32 hpf.** Genotype and treatment combinations are shown in bold text with gray highlights. Comparisons involve the distribution of the following four phenotypic classes. Truncated: maximal, moderate and, minimal. Non-truncated: Full. Significance values were calculated using two-sided Fisher’s Exact tests and significant differences (*p* < .0083; highlighted in green) assigned using a Bonferroni type adjustment for six pairwise genotype comparisons (0.05/6 = .0083). See **Figure 3-figure supplement 1**.

**Significance values (*p*) obtained by comparing the penetrance of Se-DLAV truncations (embryos with Se-DLAV truncations *vs.* embryos without Se-DLAV truncations).** See **Figure 3-figure supplement 1A.**

|  | **WT (SU5416)** | ***plxnd1^skt6^* (DMSO)** | ***plxnd1^skt6^* (SU5416)** |
| --- | --- | --- | --- |
| **WT (DMSO)** | .00003 | 0.00234 | < .0001 |
| **WT (SU5416)** |  | 0.08225 | 0.23684 |
| ***plxnd1^skt6^* (DMSO)** |  |  | 0.00371 |
| ***plxnd1^skt6^* (SU5416)** |  |  |  |

**Significance values (*p*) obtained by comparing the expressivity of Se-DLAV truncations (all four categories).** See See **Figure 3-figure supplement 1B.**

|  | **WT (SU5416)** | ***plxnd1^skt6^* (DMSO)** | ***plxnd1^skt6^* (SU5416)** |
| --- | --- | --- | --- |
| **WT (DMSO)** | < .0001 | < .0001 | < .0001 |
| **WT (SU5416)** |  | < .0001 | < .0001 |
| ***plxnd1^skt6^* (DMSO)** |  |  | < .0001 |
| ***plxnd1^skt6^* (SU5416)** |  |  |  |

**Significance values (*p*) obtained by comparing the expressivity of Se-DLAV truncations (truncated *vs.* not-truncated).** See **Figure 3-figure supplement 1B.**

|  | **WT (SU5416)** | ***plxnd1^skt6^* (DMSO)** | ***plxnd1^skt6^* (SU5416)** |
| --- | --- | --- | --- |
| **WT (DMSO)** | < .0001 | < .0001 | < .0001 |
| **WT (SU5416)** |  | 0.4586 | 0.00165 |
| ***plxnd1^skt6^* (DMSO)** |  |  | 0.00041 |
| ***plxnd1^skt6^* (SU5416)** |  |  |  |
