# Supplementary material for: GIPC proteins negatively modulate Plexind1 signaling during vascular development
Source: eLife. 2019 May 3;8:e30454. doi: 10.7554/eLife.30454 (PMC6499541; doi:10.7554/eLife.30454)
Supplement: Supplementary file 5. — Related to Figure 4B and Figure 4—figure supplement 3. [file elife-30454-supp5.docx]

**SUPPLEMENTARY FILE 5**

**Quantification of truncated Se in embryos of the indicated genotypes.** Note: the order in which the genotypes are presented here is different from that presented in Figure 4B. Related to **Figure 4B**.

| **Genotype** | **Scored Se** | | | | | | **Total embryos**  **analyzed** | **Se/embryo** |
| --- | --- | --- | --- | --- | --- | --- | --- | --- |
|  | **Truncated** | | | | **Non-truncated** | **Total Se**  **scored** |  |  |
|  | **Severe** | **Medium** | **Weak** | **Total** | **Complete** |  |  |  |
| **WT** | 0 | 0 | 1 | 1 | 137 | 138 | 12 | 11.50 |
|  | 0 % | 0 % | 0.72 % | **0.72** % | **99.28** % |  |  |  |
| ***gipc1^skt1^*** | 1 | 0 | 8 | 9 | 121 | 130 | 11 | 11.82 |
|  | 0.77 % | 0 % | 6.15 % | **6.92** % | **93.08** % |  |  |  |
| ***gipc1^skt1(MZ)^*** | 6 | 21 | 32 | 59 | 321 | 380 | 33 | 11.52 |
|  | 1.58 % | 5.53 % | 8.42 % | **15.53 %** | **84.47 %** |  |  |  |
| ***gipc2^skt3/skt4^*** | 0 | 0 | 4 | 4 | 126 | 130 | 11 | 11.82 |
|  | 0 % | 0 % | 3.08 % | **3.08 %** | **96.92 %** |  |  |  |
| ***gipc1^skt1^*; *gipc2^skt3/skt4^*** | 3 | 4 | 5 | 12 | 140 | 152 | 13 | 11.69 |
|  | 1.97 % | 2.63 % | 3.29 % | **7.89 %** | **92.11 %** |  |  |  |
| ***gipc1^skt1(MZ)^*; *gipc2^skt3/skt4^*** | 16 | 7 | 20 | 43 | 177 | 220 | 19 | 11.58 |
|  | 7.27 % | 3.18 % | 9.09 % | **19.55**  **%** | **80.45 %** |  |  |  |

**Quantification. Penetrance of Se truncations in 32 hpf embryos of the indicated genotypes.** Note: the order in which the genotypes are presented here is different from that presented in Figure 4B and Figure 4-figure supplement 3A. Related to **Figure 4-figure supplement 3A**.

| **Genotype** | **Embryos** | | |
| --- | --- | --- | --- |
|  | **With**  **Se truncations** | **Without**  **Se truncations** | **Total embryos analyzed** |
| **WT** | 1 | 11 | 12 |
|  | **8.33 %** | **91.67 %** | **100 %** |
| ***gipc1^skt1^*** | 5 | 6 | 11 |
|  | **45.45 %** | **54.55 %** | **100 %** |
| ***gipc1^skt1(MZ)^*** | 13 | 20 | 33 |
|  | **39.39 %** | **60.61 %** | **100 %** |
| ***gipc2^skt3/skt4^*** | 3 | 8 | 11 |
|  | **27.27 %** | **72.73 %** | **100 %** |
| ***gipc1^skt1^*; *gipc2^skt3/skt4^*** | 7 | 6 | 13 |
|  | **53.85 %** | **46.15 %** | **100 %** |
| ***gipc1^skt1(MZ)^*; *gipc2^skt3/skt4^*** | 13 | 6 | 19 |
|  | **68.42 %** | **31.58 %** | **100 %** |

**Quantification. Expressivity of Se truncations in 32 hpf embryos of the indicated genotypes.** Note: the order in which the genotypes are presented here is different from that presented in Figure 4B and Figure 4B. Related to **Figure 4-figure supplement 3B**.

| **Genotype** | **Se vessels in embryos with Se truncations** | | | | | | **Embryos with Se truncations** |
| --- | --- | --- | --- | --- | --- | --- | --- |
|  | **Truncated** | | | | **Non-truncated** | **Total**  **Se vessels**  **scored** |  |
|  | **Severe** | **Medium** | **Weak** | **Total** | **Complete** |  |  |
| **WT** | 0 | 0 | 1 | 1 | 9 | 10 | 1/12 |
|  | 0 % | 0 % | 10 % | **10 %** | **90 %** | 100 % |  |
| ***gipc1^skt1^*** | 1 | 0 | 8 | 9 | 51 | 60 | 5/11 |
|  | 1.67 % | 0 % | 13.33 % | **15 %** | **85 %** | 100 % |  |
| ***gipc1^skt1(MZ)^*** | 6 | 21 | 32 | 59 | 95 | 154 | 13/33 |
|  | 3.90 % | 13.64 % | 20.78 % | **38.31** % | **61.69** % | 100 % |  |
| ***gipc2^skt3/skt4^*** | 0 | 0 | 4 | 4 | 32 | 36 | 3/11 |
|  | 0 % | 0 % | 11.10 % | **11.10** % | **88.90** % | 100 % |  |
| ***gipc1^skt1^*; *gipc2^skt3/skt4^*** | 3 | 4 | 5 | 12 | 72 | 84 | 7/13 |
|  | 3.57 % | 4.76 % | 5.95 % | **14.29** % | **85.71** % | 100 % |  |
| ***gipc1^skt1(MZ)^*; *gipc2^skt3/skt4^*** | 16 | 7 | 20 | 43 | 107 | 150 | 13/19 |
|  | 10.67 % | 4.67 % | 13.33 % | **28.67** % | **71.33** % | 100 % |  |

**Significance values (*p*) obtained by comparing the distributions of Se truncations between embryos of the indicated genotypes at 32 hpf.** Genotypes are shown in bold text with gray highlights. Distributions involve the following four phenotypic classes. Truncated: severe, medium and, weak. Non-truncated: Complete. Significance values were calculated using two-sided Fisher’s Exact tests and significant differences (*p* < .0033; highlighted in green) assigned using a Bonferroni type adjustment for fifteen pairwise genotype comparisons (0.05/15 = 0.0033). Related to **Figure 4B**.

**Significance values (*p*) of pairwise comparisons of the distribution of Se truncations (all four categories).**

|  | ***gipc1^skt1^*** | ***gipc1^skt1(MZ)^*** | ***gipc2^skt3/skt4^*** | ***gipc1^skt1^*;**  ***gipc2^skt3/skt4^*** | ***gipc1^skt1(MZ)^*;**  ***gipc2^skt3/skt4^*** |
| --- | --- | --- | --- | --- | --- |
| **WT** | .00967 | < .0001 | .202 | .01905 | < .0001 |
| ***gipc1^skt1^*** |  | .00963 | .2545 | .158 | .00168 |
| ***gipc1^skt1(MZ)^*** |  |  | .00055 | .0628 | .00319 |
| ***gipc2^skt3/skt4^*** |  |  |  | .1079 | < .0001 |
| ***gipc1^skt1^*;**  ***gipc2^skt3/skt4^*** |  |  |  |  | .00934 |

**Significance values (*p*) of pairwise comparisons of the distribution of Se truncations (truncated *vs.* not-truncated).**

|  | ***gipc1^skt1^*** | ***gipc1^skt1(MZ)^*** | ***gipc2^skt3/skt4^*** | ***gipc1^skt1^*;**  ***gipc2^skt3/skt4^*** | ***gipc1^skt1(MZ)^*;**  ***gipc2^skt3/skt4^*** |
| --- | --- | --- | --- | --- | --- |
| **WT** | .00854 | < .0001 | .20198 | .00319 | < .0001 |
| ***gipc1^skt1^*** |  | .01114 | .25449 | .82281 | .00104 |
| ***gipc1^skt1(MZ)^*** |  |  | < .0001 | .02324 | .21607 |
| ***gipc2^skt3/skt4^*** |  |  |  | .11978 | < .0001 |
| ***gipc1^skt1^*;**  ***gipc2^skt3/skt4^*** |  |  |  |  | .00172 |

**Significance values (*p*) of pairwise comparisons of the distribution of Se truncations (severe *vs.* the other three categories).**

|  | ***gipc1^skt1^*** | ***gipc1^skt1(MZ)^*** | ***gipc2^skt3/skt4^*** | ***gipc1^skt1^*;**  ***gipc2^skt3/skt4^*** | ***gipc1^skt1(MZ)^*;**  ***gipc2^skt3/skt4^*** |
| --- | --- | --- | --- | --- | --- |
| **WT** | .4851 | .3493 | 1 | .2492 | .00039 |
| ***gipc1^skt1^*** |  | .6844 | 1 | .6269 | .00434 |
| ***gipc1^skt1(MZ)^*** |  |  | .3455 | .7196 | .00055 |
| ***gipc2^skt3/skt4^*** |  |  |  | .2519 | .00078 |
| ***gipc1^skt1^*;**  ***gipc2^skt3/skt4^*** |  |  |  |  | .02909 |

**Significance values (*p*) of pairwise comparisons of the distribution of Se truncations (medium *vs.* the other three categories).**

|  | ***gipc1^skt1^*** | ***gipc1^skt1(MZ)^*** | ***gipc2^skt3/skt4^*** | ***gipc1^skt1^*;**  ***gipc2^skt3/skt4^*** | ***gipc1^skt1(MZ)^*;**  ***gipc2^skt3/skt4^*** |
| --- | --- | --- | --- | --- | --- |
| **WT** | 1 | .00184 | 1 | .12416 | .04644 |
| ***gipc1^skt1^*** |  | .00341 | 1 | .1269 | .04919 |
| ***gipc1^skt1(MZ)^*** |  |  | .00341 | .17961 | .23059 |
| ***gipc2^skt3/skt4^*** |  |  |  | .1269 | .04919 |
| ***gipc1^skt1^*;**  ***gipc2^skt3/skt4^*** |  |  |  |  | .7642 |

**Significance values (*p*) of pairwise comparisons of the distribution of Se truncations (weak *vs.* the other three categories).**

|  | ***gipc1^skt1^*** | ***gipc1^skt1(MZ)^*** | ***gipc2^skt3/skt4^*** | ***gipc1^skt1^*;**  ***gipc2^skt3/skt4^*** | ***gipc1^skt1(MZ)^*;**  ***gipc2^skt3/skt4^*** |
| --- | --- | --- | --- | --- | --- |
| **WT** | .01658 | .00043 | .20199 | .21718 | .00074 |
| ***gipc1^skt1^*** |  | .45635 | .37625 | .2708 | .41625 |
| ***gipc1^skt1(MZ)^*** |  |  | .04611 | .03775 | .7657 |
| ***gipc2^skt3/skt4^*** |  |  |  | 1 | .04655 |
| ***gipc1^skt1^*;**  ***gipc2^skt3/skt4^*** |  |  |  |  | .03416 |

**Significance values (*p*) obtained by comparing the penetrance and expressivity of Se angiogenesis deficits between embryos of the indicated genotypes at 32 hpf.** Genotypes are shown in bold text with gray highlights. Distributions involve the following four phenotypic classes. Truncated: severe, medium and, weak. Non-truncated: Complete. Significance values were calculated using two-sided Fisher’s Exact tests and significant differences (*p* < .0033; highlighted in green) assigned using a Bonferroni type adjustment for fifteen pairwise genotype comparisons (0.05/15 = 0.0033). Related to **Figure 4-figure supplement 3**.

**Significance values (*p*) obtained by comparing the penetrance of Se truncations (embryos with Se truncations *vs.* embryos without Se truncations)**.

|  | ***gipc1^skt1^*** | ***gipc1^skt1(MZ)^*** | ***gipc2^skt3/skt4^*** | ***gipc1^skt1^*;**  ***gipc2^skt3/skt4^*** | ***gipc1^skt1(MZ)^*;**  ***gipc2^skt3/skt4^*** |
| --- | --- | --- | --- | --- | --- |
| **WT** | .0686 | .0700 | .3168 | .0302 | .0022 |
| ***gipc1^skt1^*** |  | .7375 | .6594 | 1 | .2663 |
| ***gipc1^skt1(MZ)^*** |  |  | .7190 | .5115 | .0828 |
| ***gipc2^skt3/skt4^*** |  |  |  | .2397 | .0567 |
| ***gipc1^skt1^*;**  ***gipc2^skt3/skt4^*** |  |  |  |  | .4735 |

**Significance values (*p*) obtained by comparing the expressivity of Se truncations (all four categories).**

|  | ***gipc1^skt1^*** | ***gipc1^skt1(MZ)^*** | ***gipc2^skt3/skt4^*** | ***gipc1^skt1^*;**  ***gipc2^skt3/skt4^*** | ***gipc1^skt1(MZ)^*;**  ***gipc2^skt3/skt4^*** |
| --- | --- | --- | --- | --- | --- |
| **WT** | 1 | .53559 | 1 | .79224 | .90613 |
| ***gipc1^skt1^*** |  | .00103 | 1 | .14342 | .03478 |
| ***gipc1^skt1(MZ)^*** |  |  | .00759 | .00054 | .00157 |
| ***gipc2^skt3/skt4^*** |  |  |  | .37995 | .07205 |
| ***gipc1^skt1^*;**  ***gipc2^skt3/skt4^*** |  |  |  |  | .05492 |

**Significance values (*p*) obtained by comparing the expressivity of Se truncations (truncated *vs.* not-truncated).**

|  | ***gipc1^skt1^*** | ***gipc1^skt1(MZ)^*** | ***gipc2^skt3/skt4^*** | ***gipc1^skt1^*;**  ***gipc2^skt3/skt4^*** | ***gipc1^skt1(MZ)^*;**  ***gipc2^skt3/skt4^*** |
| --- | --- | --- | --- | --- | --- |
| **WT** | 1 | 0.0943 | 1 | 1 | 0.2870 |
| ***gipc1^skt1^*** |  | 0.00098 | 0.7610 | 1 | 0.0507 |
| ***gipc1^skt1(MZ)^*** |  |  | 0.0015 | <0.0001 | 0.0890 |
| ***gipc2^skt3/skt4^*** |  |  |  | 0.7744 | 0.0326 |
| ***gipc1^skt1^*;**  ***gipc2^skt3/skt4^*** |  |  |  |  | 0.0155 |
