# Supplementary material for: GIPC proteins negatively modulate Plexind1 signaling during vascular development
Source: eLife. 2019 May 3;8:e30454. doi: 10.7554/eLife.30454 (PMC6499541; doi:10.7554/eLife.30454)
Supplement: Supplementary file 6. — Related to Figure 5C and Figure 5—figure supplement 1. [file elife-30454-supp6.docx]

**SUPPLEMENTARY FILE 6**

**Quantification. Percentage of Se-DLAV truncations in 32 hpf embryos** of the indicated genotypes belonging to each of the following four phenotypic classes. Truncated: maximal, moderate and, minimal. Non-truncated: Full. Related to **Figure 5C.**

| **Genotype** | **Scored Se-DLAV** | | | | | | **Total embryos**  **analyzed** | **Se-DLAV/**  **embryo** |
| --- | --- | --- | --- | --- | --- | --- | --- | --- |
|  | **Truncated DLAV** | | | | **Non-truncated**  **DLAV** | **Total Se-DLAV**  **scored** |  |  |
|  | **Maximal** | **Moderate** | **Minimal** | **Total** | **Full** |  |  |  |
| ***gipc1^skt1^*^(MZ)^**  **(siblings)** | 1 | 20 | 52 | 73 | 317 | 390 | 36 | 10.83 |
|  | 0.3 % | 5.1 % | 13.3 % | **18.7 %** | **81.3 %** |  |  |  |
| ***gipc1^skt1^*^(MZ)^; *plxnd1^fov01b/+^***  **(siblings)** | 0 | 3 | 6 | 9 | 401 | 410 | 38 | 10.79 |
|  | 0 % | 0.7 % | 1.5 % | **2.2 %** | **97.8 %** |  |  |  |

**Quantification. Penetrance of Se-DLAV truncations in 32 hpf embryos of the indicated genotypes.** Related to **Figure 5-figure supplement 1A**.

| **Genotype** | **Embryos with**  **Se-DLAV truncations** | **Embryos without**  **Se-DLAV truncations** | **Total embryos analyzed** |
| --- | --- | --- | --- |
| ***gipc1^skt1^*^(MZ)^**  **(siblings)** | 14 | 22 | 36 |
|  | **38.9 %** | **61.1 %** | **100 %** |
| ***gipc1^skt1^*^(MZ)^; *plxnd1^fov01b/+^***  **(siblings)** | 6 | 32 | 38 |
|  | **15.8 %** | **84.2 %** | **100 %** |

**Quantification. Expressivity of Se-DLAV truncations in 32 hpf embryos of the indicated genotypes.** Related to **Figure 5-figure supplement 1B**.

| **Genotype** | **Se-DLAV in embryos with Se-DLAV truncations** | | | | | | **Embryos with Se-DLAV truncations** |
| --- | --- | --- | --- | --- | --- | --- | --- |
|  | **Truncated** | | | | **Non-truncated** | **Total**  **Se-DLAV**  **scored** |  |
|  | **Maximal** | **Moderate** | **Minimal** | **Total** | **Full** |  |  |
| ***gipc1^skt1^*^(MZ)^**  **(siblings)** | 1 | 20 | 52 | 73 | 91 | 164 | 6/36 |
|  | 0.6 % | 12.2 % | 31.7 % | **44.5 %** | **55.5%** | 100 % |  |
| ***gipc1^skt1^*^(MZ)^; *plxnd1^fov01b/+^***  **(siblings)** | 0 | 3 | 6 | 9 | 57 | 66 | 2/38 |
|  | 0 % | 4.5% | 9.1% | **13.6%** | **86.4%** | 100% |  |

**Significance values (*p*) obtained by comparing the distributions of Se-DLAV truncations between *gipc1^skt1(MZ)^* and *gipc1^skt1(MZ)^*; *plxnd1^fov01b/+^* mutant siblings at 32 hpf.** Genotypes are shown in bold text with gray highlights. Distributions involve the following four phenotypic classes. Truncated: maximal, moderate and, minimal. Non-truncated: Full. Significance values were calculated using two-sided Fisher’s Exact tests, *p* < .05. Significant differences are highlighted in green*.* See **Figure 5C**.

| **Genotype pair** | | **Comparison of the distributions of Se-DLAV truncations** | | | | |
| --- | --- | --- | --- | --- | --- | --- |
|  |  | **All four categories** | **Truncated**  ***vs.***  **not-truncated** | **Maximal**  ***vs.***  **the other three categories** | **Moderate**  ***vs.***  **the other three categories** | **Minimal**  ***vs.***  **the other three categories** |
| ***gipc1^skt1(MZ)^***  **(siblings)** | ***gipc1^skt1(MZ)^*; *plxnd1^fov01b/+^***  **(siblings)** | <.0001 | <.0001 | .4875 | .00017 | <.0001 |

**Significance values (*p*) obtained by comparing the penetrance and expressivity of Se-DLAV angiogenesis deficits between *gipc1^skt1(MZ)^* and *gipc1^skt1(MZ)^*; *plxnd1^fov01b/+^* mutant siblings at 32 hpf.** Genotypes are shown in bold text with gray highlights. Comparisons involve the distribution of the following four phenotypic classes. Truncated: maximal, moderate and, minimal. Non-truncated: Full. Significance values were calculated using two-sided Fisher’s Exact tests, *p* < .05. Significant differences are highlighted in green*.* See **Figure 5-figure supplement 1**.

|  | | **Comparisons** | | |
| --- | --- | --- | --- | --- |
|  | | **Penetrance** | **Expressivity** | |
| **Genotype pair** | | **Truncated**  ***vs.***  **not-truncated** | **All four categories** | **Truncated**  ***vs.***  **not-truncated** |
| ***gipc1^skt1(MZ)^***  **(siblings)** | ***gipc1^skt1(MZ)^*; *plxnd1^fov01b/+^***  **(siblings)** | .0361 | .00004 | .0009 |
