# Supplementary material for: GIPC proteins negatively modulate Plexind1 signaling during vascular development
Source: eLife. 2019 May 3;8:e30454. doi: 10.7554/eLife.30454 (PMC6499541; doi:10.7554/eLife.30454)
Supplement: Supplementary file 7. [file elife-30454-supp7.docx]

**SUPPLEMENTARY FILE 7**

**Raw densitometry values for pERK in Western Blots of relative ERK activity experiments.** Conditions. Control (bold black font), *PLXND1* loss (bold red font), *GIPC* loss (bold green font) and, *GIPC-PLXND1* double loss (bold blue font). Related to **Figure 7E** and **Figure 7-figure supplement 1**.

| **Conditions** | | **Independent experiment** | **Treatment** | | |
| --- | --- | --- | --- | --- | --- |
| **shRNA** | **gRNA** |  | **Vehicle** | **2 nM SEMA3E** | |
|  |  |  |  | **15 min** | **45 min** |
| **Non-targeting** | **Non-targeting gRNA #1** | **1** | 31.7 | 9.1 | 12.5 |
|  |  | **2** | 23.5 | 8.5 | 8.1 |
|  |  | **3** | 33.3 | 13.1 | 17.2 |
|  |  | **4** | 32.4 | 27.3 | 25.9 |
|  | **Non-targeting gRNA #2** | **1** | 52.5 | 26.6 | 23.7 |
|  |  | **2** | 43.8 | 8.2 | 17.4 |
|  |  | **3** | 44.0 | 27.6 | 33.0 |
|  |  | **4** | 47.0 | 30.5 | 19.7 |
| **Non-targeting** | ***PLXND1* gRNA KO #1** | **1** | 66.0 | 76.1 | 67.0 |
|  |  | **2** | 75.9 | 91.1 | 60.9 |
|  |  | **3** | 36.3 | 37 | 45.6 |
|  |  | **4** | 73.9 | 57.4 | 56.3 |
|  | ***PLXND1* gRNA KO #2** | **1** | 54.4 | 58.3 | 46.5 |
|  |  | **2** | 60.5 | 60.8 | 44.2 |
|  |  | **3** | 55.6 | 54.8 | 50.5 |
|  |  | **4** | 64.3 | 59.5 | 66.6 |
| ***GIPC*s** | **Non-targeting gRNA #1** | **1** | 41.3 | 9.3 | 8.0 |
|  |  | **2** | 29.7 | 3.4 | 2.9 |
|  |  | **3** | 32.3 | 22.4 | 9.5 |
|  |  | **4** | 31.3 | 18.4 | 6.3 |
|  | **Non-targeting gRNA #2** | **1** | 26.0 | 2.7 | 11.5 |
|  |  | **2** | 28.2 | 8.5 | 6.9 |
|  |  | **3** | 51.1 | 25.3 | 8.5 |
|  |  | **4** | 61.7 | 33.2 | 13.8 |
| ***GIPC*s** | ***PLXND1* gRNA KO #1** | **1** | 61.0 | 60.6 | 42.7 |
|  |  | **2** | 55.0 | 53.7 | 43.9 |
|  |  | **3** | 33.3 | 35.4 | 31.0 |
|  |  | **4** | 58.3 | 57 | 58.6 |
|  | ***PLXND1* gRNA KO #2** | **1** | 43.6 | 36.1 | 31.8 |
|  |  | **2** | 62.9 | 57.2 | 51.2 |
|  |  | **3** | 60.1 | 57.5 | 47.3 |
|  |  | **4** | 67.2 | 63.0 | 64.0 |

**Raw densitometry values for ERK_Total_ in Western Blots of relative ERK activity experiments.** Conditions. Control (bold black font), *PLXND1* loss (bold red font), *GIPC* loss (bold green font) and, *GIPC-PLXND1* double loss (bold blue font). Related to **Figure 7E** and **Figure 7-figure supplement 1**.

| **Conditions** | | **Independent experiment** | **Treatment** | | |
| --- | --- | --- | --- | --- | --- |
| **shRNA** | **gRNA** |  | **Vehicle** | **2 nM SEMA3E** | |
|  |  |  |  | **15 min** | **45 min** |
| **Non-targeting** | **Non-targeting gRNA #1** | **1** | 77.3 | 86.8 | 93.5 |
|  |  | **2** | 69.3 | 72.6 | 84.0 |
|  |  | **3** | 60.3 | 59.1 | 80.4 |
|  |  | **4** | 78.4 | 80.5 | 93.9 |
|  | **Non-targeting gRNA #2** | **1** | 74.8 | 81.1 | 91.4 |
|  |  | **2** | 96.8 | 87.6 | 91.8 |
|  |  | **3** | 88.5 | 88.5 | 100.1 |
|  |  | **4** | 86.6 | 93.5 | 104.0 |
| **Non-targeting** | ***PLXND1* gRNA KO #1** | **1** | 67.1 | 83.1 | 81.5 |
|  |  | **2** | 58.6 | 79.8 | 73.0 |
|  |  | **3** | 47.6 | 53.2 | 53.7 |
|  |  | **4** | 78.2 | 77.6 | 81.4 |
|  | ***PLXND1* gRNA KO #2** | **1** | 99.6 | 108.1 | 109.6 |
|  |  | **2** | 106.1 | 105.5 | 87.6 |
|  |  | **3** | 93.7 | 104.2 | 111.3 |
|  |  | **4** | 86.6 | 93.5 | 104.0 |
| ***GIPC*s** | **Non-targeting gRNA #1** | **1** | 67.7 | 73.8 | 73.1 |
|  |  | **2** | 76.5 | 77.0 | 82.9 |
|  |  | **3** | 47.0 | 51.6 | 65.9 |
|  |  | **4** | 63.0 | 82.9 | 77.0 |
|  | **Non-targeting gRNA #2** | **1** | 83.3 | 96.2 | 99.2 |
|  |  | **2** | 98.9 | 102.3 | 90.5 |
|  |  | **3** | 111.7 | 111.0 | 118.2 |
|  |  | **4** | 99.8 | 110.8 | 109.2 |
| ***GIPC*s** | ***PLXND1* gRNA KO #1** | **1** | 66.4 | 72.0 | 80.4 |
|  |  | **2** | 74.0 | 70.1 | 78.5 |
|  |  | **3** | 46.9 | 49.4 | 49.2 |
|  |  | **4** | 69.7 | 74.4 | 81.3 |
|  | ***PLXND1* gRNA KO #2** | **1** | 95.3 | 92.5 | 80.3 |
|  |  | **2** | 97.6 | 87.4 | 94.1 |
|  |  | **3** | 111.5 | 120.6 | 105.7 |
|  |  | **4** | 97.3 | 109.4 | 115.4 |

**Average densitometry values for pERK and ERK_Total_ and relative ERK activity (average pERK/average ERK_Total_) values for cells in the indicated conditions.** Conditions. Control (bold black font), *PLXND1* loss (bold red font), *GIPC* loss (bold green font) and, *GIPC-PLXND1* double loss (bold blue font). Relative ERK activities for each treatment were normalized to the values observed in the corresponding vehicle treatment. Related to **Figure 7E** and **Figure 7-figure supplement 1**.

**Values for cells in the described conditions.** Related to **Figure 7E**.

| **Conditions (pools)** | |  |  | **Treatment** | | |
| --- | --- | --- | --- | --- | --- | --- |
| **shRNA** | **gRNA** |  |  | **Vehicle** | **2 nM SEMA3E** | |
|  |  |  |  |  | **15 min** | **45 min** |
| **Non-targeting** | **Non-targeting gRNAs**  **(#1 and #2 data pool)** | **Average**  **densitometry** | pERK | 38.5 | 18.9 | 19.7 |
|  |  |  | ERK_Total_ | 79 | 81.2 | 92.4 |
|  |  | **Relative ERK activity** | **Ratio** | 0.488 | 0.232 | 0.213 |
|  |  |  | **Percentage** | **100 %** | **47.6 %** | **43.7 %** |
| **Non-targeting** | ***PLXND1* gRNA KOs**  **(#1 and #2 data pool)** | **Average**  **densitometry** | pERK | 60.9 | 61.9 | 54.7 |
|  |  |  | ERK_Total_ | 79.69 | 88.1 | 87.8 |
|  |  | **Relative ERK activity** | **Ratio** | 0.764 | 0.702 | 0.623 |
|  |  |  | **Percentage** | **100 %** | **91.9 %** | **81.6 %** |
| ***GIPC*s** | **Non-targeting gRNAs**  **(#1 and #2 data pool)** | **Average**  **densitometry** | pERK | 37.7 | 15.4 | 8.4 |
|  |  |  | ERK_Total_ | 81.0 | 88.2 | 89.5 |
|  |  | **Relative ERK activity** | **Ratio** | 0.466 | 0.175 | 0.094 |
|  |  |  | **Percentage** | **100 %** | **37.5 %** | **20.2 %** |
| ***GIPC*s** | ***PLXND1* gRNAs KOs**  **(#1 and #2 data pool)** | **Average**  **densitometry** | pERK | 55.2 | 52.6 | 46.3 |
|  |  |  | ERK_Total_ | 82.3 | 84.5 | 85.6 |
|  |  | **Relative ERK activity** | **Ratio** | 0.670 | 0.622 | 0.541 |
|  |  |  | **Percentage** | **100 %** | **92.9 %** | **80.7 %** |

**Values for cells in conditions involving non-targeting gRNA #1 and *PLXND1* gRNA KO #1.** Related to **Figure 7-figure supplement 1A**.

| **Conditions** | |  |  | **Treatment** | | |
| --- | --- | --- | --- | --- | --- | --- |
| **shRNA** | **gRNA** |  |  | **Vehicle** | **2 nM SEMA3E** | |
|  |  |  |  |  | **15 min** | **45 min** |
| **Non-targeting** | **Non-targeting gRNA #1** | **Average**  **densitometry** | pERK | 30.2 | 14.5 | 15.9 |
|  |  |  | ERK_Total_ | 71.3 | 74.8 | 87.95 |
|  |  | **Relative ERK activity** | **Ratio** | 0.424 | 0.194 | 0.181 |
|  |  |  | **Percentage** | **100 %** | **45.7%** | **42.7%** |
| **Non-targeting** | ***PLXND1* gRNA KO #1** | **Average**  **densitometry** | pERK | 63.0 | 65.4 | 57.45 |
|  |  |  | ERK_Total_ | 62.9 | 73.4 | 72.4 |
|  |  | **Relative ERK activity** | **Ratio** | 1.002 | 0.891 | 0.794 |
|  |  |  | **Percentage** | **100 %** | **88.9 %** | **79.2 %** |
| ***GIPC*s** | **Non-targeting gRNA #1** | **Average**  **densitometry** | pERK | 33.65 | 13.4 | 6.7 |
|  |  |  | ERK_Total_ | 63.55 | 71.3 | 74.7 |
|  |  | **Relative ERK activity** | **Ratio** | 0.530 | 0.188 | 0.089 |
|  |  |  | **Percentage** | **100 %** | **35.4 %** | **16.9 %** |
| ***GIPC*s** | ***PLXND1* gRNA KO #1** | **Average**  **densitometry** | pERK | 51.9 | 51.7 | 44.05 |
|  |  |  | ERK_Total_ | 64.25 | 66.5 | 72.35 |
|  |  | **Relative ERK activity** | **Ratio** | 0.808 | 0.777 | 0.609 |
|  |  |  | **Percentage** | **100 %** | **96.2 %** | **75.4 %** |

**Values for cells in conditions involving non-targeting gRNA #2 and *PLXND1* gRNA KO #2.** Related to **Figure 7-figure supplement 1B**.

| **Conditions** | |  |  | **Treatment** | | |
| --- | --- | --- | --- | --- | --- | --- |
| **shRNA** | **gRNA** |  |  | **Vehicle** | **2 nM SEMA3E** | |
|  |  |  |  |  | **15 min** | **45 min** |
| **Non-targeting** | **Non-targeting gRNA #2** | **Average**  **densitometry** | pERK | 46.8 | 23.2 | 23.45 |
|  |  |  | ERK_Total_ | 86.7 | 87.7 | 96.8 |
|  |  | **Relative ERK activity** | **Ratio** | 0.540 | 0.265 | 0.242 |
|  |  |  | **Percentage** | **100 %** | **49.1 %** | **44.8 %** |
| **Non-targeting** | ***PLXND1* gRNA KO #2** | **Average**  **densitometry** | pERK | 58.7 | 58.35 | 51.95 |
|  |  |  | ERK_Total_ | 96.5 | 102.825 | 103.125 |
|  |  | **Relative ERK activity** | **Ratio** | 0.608 | 0.567 | 0.504 |
|  |  |  | **Percentage** | **100 %** | **93.3 %** | **82.9 %** |
| ***GIPC*s** | **Non-targeting gRNA #2** | **Average**  **densitometry** | pERK | 41.75 | 17.4 | 10.2 |
|  |  |  | ERK_Total_ | 98.4 | 105.1 | 104.3 |
|  |  | **Relative ERK activity** | **Ratio** | 0.424 | 0.166 | 0.098 |
|  |  |  | **Percentage** | **100 %** | **39.1 %** | **23.0 %** |
| ***GIPC*s** | ***PLXND1* gRNA KO #2** | **Average**  **densitometry** | pERK | 58.45 | 53.45 | 48.6 |
|  |  |  | ERK_Total_ | 100.4 | 102.5 | 98.9 |
|  |  | **Relative ERK activity** | **Ratio** | 0.582 | 0.522 | 0.491 |
|  |  |  | **Percentage** | **100 %** | **89.6 %** | **84.4 %** |

**Statistical significances**. One-way ANOVA tests, followed by Tukey post hoc analyses, were conducted to determine if relative ERK activity was significantly different (p < .05) between conditions across treatments. Conditions. Control (bold black font), *PLXND1* loss (bold red font), *GIPC* loss (bold green font) and, *GIPC-PLXND1* double loss (bold blue font). n = 4 independent experiments per *PLXND1* gRNA KO (for a pooled total of 8 experiments); n = 4 independent experiments per non-targeting gRNA (for a pooled total of 8 experiments). Pairs of conditions (pools) with significantly different relative ERK activity under the same treatment are highlighted in green. Related to **Figure 7E** and **Figure 7-figure supplement 1**.

| **Conditions (pools)** | | **Treatments** | | |
| --- | --- | --- | --- | --- |
|  |  | **Vehicle** | **2 nm SEMA3E** | |
|  |  |  | **15 min** | **45 min** |
| **Non-targeting shRNA,**  **non-targeting gRNAs**  **(#1 and #2 data pool)** | **Non-targeting shRNA,**  ***PLXND1* gRNAs KOs**  **(#1 and #2 data pool)** | 1 | .011 | < .0005 |
| **Non-targeting shRNA,**  **non-targeting gRNAs**  **(#1 and #2 data pool)** | ***GIPC* shRNAs,**  **Non-targeting gRNAs**  **(#1 and #2 data pool)** | 1 | .887 | .028 |
| **Non-targeting shRNA,**  **non-targeting gRNAs**  **(#1 and #2 data pool)** | ***GIPC* shRNAs,**  ***PLXND1* gRNAs KOs**  **(#1 and #2 data pool)** | 1 | .005 | < .0005 |
| **Non-targeting shRNA,**  ***PLXND1* gRNAs KOs**  **(#1 and #2 data pool)** | ***GIPC* shRNAs,**  **Non-targeting gRNAs**  **(#1 and #2 data pool)** | 1 | .002 | < .0005 |
| **Non-targeting shRNA,**  ***PLXND1* gRNAs KOs**  **(#1 and #2 data pool)** | ***GIPC* shRNAs,**  ***PLXND1* gRNAs KOs**  **(#1 and #2 data pool)** | 1 | .989 | .995 |
| ***GIPC* shRNAs,**  **Non-targeting gRNAs**  **(#1 and #2 data pool)** | ***GIPC* shRNAs,**  ***PLXND1* gRNAs KOs**  **(#1 and #2 data pool)** | 1 | .001 | < .0005 |
