# Supplementary material for: GIPC proteins negatively modulate Plexind1 signaling during vascular development
Source: eLife. 2019 May 3;8:e30454. doi: 10.7554/eLife.30454 (PMC6499541; doi:10.7554/eLife.30454)
Supplement: Supplementary file 8. [file elife-30454-supp8.docx]

**PSUPPLEMENTARY FILE 8**

**A. Sequences of the epitope-tagged proteins used for exogenous expression in mammalian cells and zebrafish embryos**. Motifs and domains as follows: V5 tag (red), FLAG tag (purple), 2xHA tag (yellow with underlining), signal peptide (bold text), GBM (magenta), GH1 (blue), PDZ (green), GH2 (gray). Related to **Figure 1, Supplementary File 1** and, **Supplementary File 2**.

**V5-C-mPLXND1^WT^** (661 aa; plasmid ID #862)

MGKPIPNPLLGLDSTENLYFQGPDPSTNSADITSLYKKAGSEFALQMEEMESQIREEISKGFAELQTDMTDLTKELNRSQGIPFLEYKHFVTRTFFPKCSSLYEERYVLPSKTLNSQGGSPPQETHPLLGEWNIPEHCRPSMEEGISLFSSLLNNKHFLIVFVHALEQQKDFAVRDRCSLASLLTIALHGKLEYYTSIMKELLVDLIDASAAKNPKLMLRRTESVVEKMLTNWMSICMYGCLRETVGEPFFLLLCAIKQQINKGSIDAITGKARYTLNEEWLLRENIEAKPRNLNVSFQGCGMDSLSVRAMDTDTLTQVKEKILEAFCKNVPYSQWPRAEDVDLEWFASSTQSYVLRDLDDTSVVEDGRKKLNTLAHYKIPEGASLAMSLTDKKDSTLGRVKDLDTEKYFHLVLPTDELVEPKKSHRQSHRKKVLPEIYLTRLLSTKGTLQKFLDDLFKAILSIREDKPPLAVKYFFDFLEEQAEKRGISDPDTLHIWKTNSLPLRFWVNILKNPQFVFDIEKTDHIDACLSVIAQAFIDACSISDLQLGKDSPTNKLLYAKEIPEYRKTVQRYYKQIQDMTPLSEQEMNAHLAEESRKYQNEFNTNVAMAEIYKYAKRYRPQIMAALEANPTARRTQLQHKFEQVVALMENNIYECYSEA*

**V5-C-mPLXND1Δ^CYSEA^** (656 aa; plasmid ID #863)

MGKPIPNPLLGLDSTENLYFQGPDPSTNSADITSLYKKAGSEFALQMEEMESQIREEISKGFAELQTDMTDLTKELNRSQGIPFLEYKHFVTRTFFPKCSSLYEERYVLPSKTLNSQGGSPPQETHPLLGEWNIPEHCRPSMEEGISLFSSLLNNKHFLIVFVHALEQQKDFAVRDRCSLASLLTIALHGKLEYYTSIMKELLVDLIDASAAKNPKLMLRRTESVVEKMLTNWMSICMYGCLRETVGEPFFLLLCAIKQQINKGSIDAITGKARYTLNEEWLLRENIEAKPRNLNVSFQGCGMDSLSVRAMDTDTLTQVKEKILEAFCKNVPYSQWPRAEDVDLEWFASSTQSYVLRDLDDTSVVEDGRKKLNTLAHYKIPEGASLAMSLTDKKDSTLGRVKDLDTEKYFHLVLPTDELVEPKKSHRQSHRKKVLPEIYLTRLLSTKGTLQKFLDDLFKAILSIREDKPPLAVKYFFDFLEEQAEKRGISDPDTLHIWKTNSLPLRFWVNILKNPQFVFDIEKTDHIDACLSVIAQAFIDACSISDLQLGKDSPTNKLLYAKEIPEYRKTVQRYYKQIQDMTPLSEQEMNAHLAEESRKYQNEFNTNVAMAEIYKYAKRYRPQIMAALEANPTARRTQLQHKFEQVVALMENNIYE*

**V5-C-m PLXND1Δ^GBM^** (652 aa; plasmid ID #1774)

MGKPIPNPLLGLDSTENLYFQGPDPSTNSADITSLYKKAGSEFALQMEEMESQIREEISKGFAELQTDMTDLTKELNRSQGIPFLEYKHFVTRTFFPKCSSLYEERYVLPSKTLNSQGGSPPQETHPLLGEWNIPEHCRPSMEEGISLFSSLLNNKHFLIVFVHALEQQKDFAVRDRCSLASLLTIALHGKLEYYTSIMKELLVDLIDASAAKNPKLMLRRTESVVEKMLTNWMSICMYGCLRETVGEPFFLLLCAIKQQINKGSIDAITGKARYTLNEEWLLRENIEAKPRNLNVSFQGCGMDSLSVRAMDTDTLTQVKEKILEAFCKNVPYSQWPRAEDVDLEWFASSTQSYVLRDLDDTSVVEDGRKKLNTLAHYKIPEGASLAMSLTDKKDSTLGRVKDLDTEKYFHLVLPTDELVEPKKSHRQSHRKKVLPEIYLTRLLSTKGTLQKFLDDLFKAILSIREDKPPLAVKYFFDFLEEQAEKRGISDPDTLHIWKTNSLPLRFWVNILKNPQFVFDIEKTDHIDACLSVIAQAFIDACSISDLQLGKDSPTNKLLYAKEIPEYRKTVQRYYKQIQDMTPLSEQEMNAHLAEESRKYQNEFNTNVAMAEIYKYAKRYRPQIMAALEANPTARRTQLQHKFEQVVALMEN*

**FLAG-mGIPC1^WT^** (344 aa; plasmid ID #864)

MDYKDDDDKLEMPLGLGRRKKAPPLVENEEAEPSRSGLGVGEPGPLGGSGAGESQMGLPPPPASLRPRLVFHTQLAHGSPTGRIEGFTNVKELYGKIAEAFRLPAAEVMFCTLNTHKVDMDKLLGGQIGLEDFIFAHVKGQRKEVEVFKSEDALGLTITDNGAGYAFIKRIKEGSVIDHIQLISVGDMIEAINGQSLLGCRHYEVARLLKELPRGRTFTLKLTEPRKAFDMISQRSAGGHPGSGPQLGTGRGTLRLRSRGPATVEDLPSAFEEKAIEKVDDLLESYMGIRDTELAATMVELGKDKRNPDELAEALDERLGDFAFPDEFVFDVWGAIGDAKVGRY*

**FLAG-mGIPC1^GH1^** (135 aa; plasmid ID #868)

MDYKDDDDKLEMPLGLGRRKKAPPLVENEEAEPSRSGLGVGEPGPLGGSGAGESQMGLPPPPASLRPRLVFHTQLAHGSPTGRIEGFTNVKELYGKIAEAFRLPAAEVMFCTLNTHKVDMDKLLGGQIGLEDFIF*

**FLAG-mGIPC1^PDZ^** (110 aa; plasmid ID #866)

MDYKDDDDKAHVKGQRKEVEVFKSEDALGLTITDNGAGYAFIKRIKEGSVIDHIQLISVGDMIEAINGQSLLGCRHYEVARLLKELPRGRTFTLKLTEPRKAFDMISQRS*

**Zebrafish 2xHA-Plxnd1^WT^** (1902 aa; plasmid ID #1414)

**MPPSAADRRSALALLSALLLAALQTRSALA**LHVQYPYDVPDYAYPYDVPDYALHVQQAFATPGRTNNFALDAASGRVYLAAVNNLYQLNATLALEVEMRTGPVLDNPLCHAPQLPQATCEHQKTLTDNHNKLLALDRAQDVLLACGSVYQGFCELRRLENVSRLAVQFPQDGATVFPSMLNIAANHENASTVGLVFRTHGGSPRLLVGATYTGMGTEYFPKNHSKEDLRFENTPEIAIRALDTRELGRLFTYDINPSEDNVFKIKQEVKQKNKLSFVHAFALGNYSYIAFNNDANSGLKESQPNSVLARICLDTDAPRRAAESRKLTESYAQMGLRCGTYTRLLSVSPAELRAETFLFAVFGRADGRAAVCVFRVAEVEEMIRQGRRNCSHGPNSDVQVLDSVIQGSGAECEGKGSIMLQLQTDQLNCGAAHLQHPLALRRPLRARPLYEAQGLSSVAVDSAHNHTLMFLGSRGRLHKVSLHSNFSVSQQWSLRLPANEPVHHIMTFDPSDRTYLYVMTSHHLLRVRVSSCEQYSSCGDCLSAGDPHCGWCTLERRCSVQQDCSSVSLSRSWISISEGVQQCPSMTITPAEISRSAEMRDVGILVAGSVPDLQGLRVECDYGMGISTNATVHLDYGTSHIQTCPLPPAHTLPTIPSGTDHVTVPVSINANGVSVVSGRFIIYDCERTGQIHSTTACTSCLSSAWRCFWDPQLHQCVSSKNNTQQLLQNSSSCPSMVAYEVPPLPTGSSPVFSLELKNVEHGTALECVYSDGQQYRAQWLDGPWVNCSGATLKTTQWSESFSLNLRRAGDATYIDNPQRMTVEVYSCSAGVSDCSQCLGRAALGQECVWCEHICRQQDQCPTTSSARCPAPLIHKIEPLSGPLTGGTLLTVTGKNLGHRADQLSVSIGGVTCHTLPQQYTVSIRLVCETDASLEQMSGQVTVGVAGDAVGVSKEQFSFVEPRLLRFSPAQGPLAGGTTLTIQGQFLDAGSTTDIQINNTHTCSIRTRSSEIIRCVMPAAASAENVSVCVVFDGRPCVSASPNFTFSYQKNPTISHIWPSRSYISGGRSISVSGVGFDLVQSVVMEVPGVGQTNCSCVSSTLILCQSPAADQAQQTAVLFSLNGVQYRDAASAGLEEGEQPHTQRYSFQYVEDPQFYTANKEKLIKHHPGEPLILIINKGPSDLDLTLDEYSVTIGSDLCDITFHNQQLFHCSINRSLSASTGELPVTVRVGHFQKVIAMVQMGGGSELAIVVSIVVCCVLLLLCTVALVVYCTKSRRAERYWQKTLVQMEEMESQIRDEIRKGFAELQTDMTDLTKELSRTQGIPFLEYKQFVTRTFFPKMCCDYERRLVQPVYENDPLGPRAHSETHPLLQDWQPAGSVRPNLEEGITLFSTLLNNKHFLVTFVHALEQQRDFAVRDRCNLASLLTIALHGKLEYYTSIMKDLLVDLIDASATKNPKLMLRRTESVVEKMLTNWMSICMYSYLKETVGEPFFLLLCAIKQQINKGSIDVLTGKARYTLNEEWLLRENIEAKPQNVMVSFQGLGMDSVCVRVMNSDTICQVKEKILEAFYKNLPYSQWPREEDVELEWFPEGRSSRILQDLDDSSVMEDGRKKLNTVFHYQIPDGASLAMSLKDKRENTLGRVKDLDTEKYVHLVLPHDELMESRKTHRQSQRKKVLPEIYLTRLLSTKGTLQKFLDDLFQAILSVPAENPPLAIKYFFDFLEEQADKRGITDPDTLHIWKTNSLPLRFWVNILKNPQFVFDIEKTDHMDACLSVIAQAFIDACSLSDLQLGKDSPTNKLLYAKEIPEYKKRVQSFYRQIQELPALSEQEMNTHLAEESRKHRNEFNTTLALTEVYKYAKRYRAQVASALDSNPTARRTQLQHKFEQVIALVEDNIYECSSEA*

**Zebrafish 2xHA-Plxnd1Δ^GBM^** (1893 aa; plasmid ID #1685)

**MPPSAADRRSALALLSALLLAALQTRSALA**LHVQYPYDVPDYAYPYDVPDYALHVQQAFATPGRTNNFALDAASGRVYLAAVNNLYQLNATLALEVEMRTGPVLDNPLCHAPQLPQATCEHQKTLTDNHNKLLALDRAQDVLLACGSVYQGFCELRRLENVSRLAVQFPQDGATVFPSMLNIAANHENASTVGLVFRTHGGSPRLLVGATYTGMGTEYFPKNHSKEDLRFENTPEIAIRALDTRELGRLFTYDINPSEDNVFKIKQEVKQKNKLSFVHAFALGNYSYIAFNNDANSGLKESQPNSVLARICLDTDAPRRAAESRKLTESYAQMGLRCGTYTRLLSVSPAELRAETFLFAVFGRADGRAAVCVFRVAEVEEMIRQGRRNCSHGPNSDVQVLDSVIQGSGAECEGKGSIMLQLQTDQLNCGAAHLQHPLALRRPLRARPLYEAQGLSSVAVDSAHNHTLMFLGSRGRLHKVSLHSNFSVSQQWSLRLPANEPVHHIMTFDPSDRTYLYVMTSHHLLRVRVSSCEQYSSCGDCLSAGDPHCGWCTLERRCSVQQDCSSVSLSRSWISISEGVQQCPSMTITPAEISRSAEMRDVGILVAGSVPDLQGLRVECDYGMGISTNATVHLDYGTSHIQTCPLPPAHTLPTIPSGTDHVTVPVSINANGVSVVSGRFIIYDCERTGQIHSTTACTSCLSSAWRCFWDPQLHQCVSSKNNTQQLLQNSSSCPSMVAYEVPPLPTGSSPVFSLELKNVEHGTALECVYSDGQQYRAQWLDGPWVNCSGATLKTTQWSESFSLNLRRAGDATYIDNPQRMTVEVYSCSAGVSDCSQCLGRAALGQECVWCEHICRQQDQCPTTSSARCPAPLIHKIEPLSGPLTGGTLLTVTGKNLGHRADQLSVSIGGVTCHTLPQQYTVSIRLVCETDASLEQMSGQVTVGVAGDAVGVSKEQFSFVEPRLLRFSPAQGPLAGGTTLTIQGQFLDAGSTTDIQINNTHTCSIRTRSSEIIRCVMPAAASAENVSVCVVFDGRPCVSASPNFTFSYQKNPTISHIWPSRSYISGGRSISVSGVGFDLVQSVVMEVPGVGQTNCSCVSSTLILCQSPAADQAQQTAVLFSLNGVQYRDAASAGLEEGEQPHTQRYSFQYVEDPQFYTANKEKLIKHHPGEPLILIINKGPSDLDLTLDEYSVTIGSDLCDITFHNQQLFHCSINRSLSASTGELPVTVRVGHFQKVIAMVQMGGGSELAIVVSIVVCCVLLLLCTVALVVYCTKSRRAERYWQKTLVQMEEMESQIRDEIRKGFAELQTDMTDLTKELSRTQGIPFLEYKQFVTRTFFPKMCCDYERRLVQPVYENDPLGPRAHSETHPLLQDWQPAGSVRPNLEEGITLFSTLLNNKHFLVTFVHALEQQRDFAVRDRCNLASLLTIALHGKLEYYTSIMKDLLVDLIDASATKNPKLMLRRTESVVEKMLTNWMSICMYSYLKETVGEPFFLLLCAIKQQINKGSIDVLTGKARYTLNEEWLLRENIEAKPQNVMVSFQGLGMDSVCVRVMNSDTICQVKEKILEAFYKNLPYSQWPREEDVELEWFPEGRSSRILQDLDDSSVMEDGRKKLNTVFHYQIPDGASLAMSLKDKRENTLGRVKDLDTEKYVHLVLPHDELMESRKTHRQSQRKKVLPEIYLTRLLSTKGTLQKFLDDLFQAILSVPAENPPLAIKYFFDFLEEQADKRGITDPDTLHIWKTNSLPLRFWVNILKNPQFVFDIEKTDHMDACLSVIAQAFIDACSLSDLQLGKDSPTNKLLYAKEIPEYKKRVQSFYRQIQELPALSEQEMNTHLAEESRKHRNEFNTTLALTEVYKYAKRYRAQVASALDSNPTARRTQLQHKFEQVIALVED*****

**B. Zebrafish Gipc and Plxnd1 proteins encoded by the WT and mutant alleles**. Residues in the mutant protein that differ from the WT protein are indicated in red. Related to **Figure 2A-B, Figure 4-figure supplement 1** and, **Supplementary File 1**.

**Zebrafish Gipc1** (335 aa; wild-type; NCBI Reference Sequence: NP_001004018.1)

MPLGLGRRKKASPLVENEEAEPIRAGLNVSGLDGLDGGRVGLGEGAAHEGLPPPPTSLRPRLIFHTQLAHGSPTGRIEGFSNVRELYAKIGEAFGIPPTEVMFCTLNTHKVDMDKLLGGQIGLEDFIFAHVKGQRKEVEVFKGEDALGLTITDNGAGYAFIKRIREGSIIHQIQVINVGDMIESINGQILIGCRHYEVAKMLKELPKGKTFFLKMVEPLKAFDMISQRSGSRSGSAQLGTGRGTLRLRSKGPATVEELPSAFEEKAIEKVDDLLESYMGIRDSELAATMVELGKDKKNPDEFAEALDETLGDFAFPDEFVFDVWGAIGDAKVGRV*

**Zebrafish Gipc1^skt1^** (25 aa; mutant)

MPLGLGRRKKASLSRTRRLNPSGLD*

**Zebrafish Gipc2** (328 aa; wild-type; NCBI Reference Sequence: NP_957224.1)

MPLGLRKKKNKSRESSNLVENEEIGGHAVVGKSAVNGGGLPPPPASLRPKLVFHTQLAHGSPTGRIEGFTNVKELYNKIAEAFNLSPDEILFCTLNTHKIDMEKLLGGQIGLEDFIFAHIKGIKKEMDVYKSEEALGLTITDNGAGYAFIKRIKEGSVVDGVKVICVGDHIECINGKNIVGMRHYEVARMLKELPKDQTFTLKLVEPMKAFEMLEPRSRGAGGKTSGEGKIGTGRETLRLRSKGPATVEEMPTEFEEKAVKKVDDLLESYMGIRDTELAATMVEVGRDKKNPDEFAMALDQALGDFAFPDEFVFDVWGAIGDAKQGRF*

**Zebrafish Gipc2^skt3^** (63 aa; mutant)

MPLGLRKKKNKSRESSNLVENEEIGGGLLPACGRNWCSIRSSRTGVPQGESRASRTSKSCTTK*

**Zebrafish Gipc2^skt4^** (82 aa; mutant)

MPLGLRKKKNKSRESSNLVENEETCRRTCSRRTCSCREVRGEVLQPAAETGVPYAARARESHRENRGLHERQRAVQQNSRSL*

**Zebrafish Gipc3** (333 aa; wild-type; NCBI Reference Sequence: NP_001038783.1)

MDAQMQQVSQNLQPMQNGEAMSPGPQDSTGPPGDEESQSTVPSAPPLPPDAAPCPRPKLVFHTQLAHGSPTGRIHGFTNVRELYAKIAEVFNISPSEILFCTLNSHKVDMQKLLGGQIGLEDFIFAHVRGETKEVEVIKTEDALGLTITDNGAGYAFIKRIKEGSTIDRIKSVCVGDHIEAINDQSIVGCRHYEVAKMLKEQPRGTPFTLRLVEPKKAFDMIGQRTRAPKSSEGKLSSGKETLRLRTRGSASLEEIPSELEDAAIRKVDDLLESYMGIRDLELATTIVEAGKNKKNPDDFAEALDSVLGDFGFPDVFLFDVWGALGDVKNGRL*

**Zebrafish Gipc3^skt5^** (21 aa; mutant)

MDAQTLSRSPRTSSPCRTERP*

**Zebrafish Plxnd1** (1880 aa; wild-type; NCBI Reference Sequence: NP_991260.2)

MPPSAADRRSALALLSALLLAALQTRSAGALHVQQAFATPGRTNNFALDAASGRVYLAAVNNLYQLNATLALEVEMRTGPVLDNPLCHAPQLPQATCEHQKTLTDNHNKLLALDRAQDVLLACGSVYQGFCELRRLENVSRLAVQFPQDGATVFPSMLNIAANHENASTVGLVFRTHGGSPRLLVGATYTGMGTEYFPKNHSKEDLRFENTPEIAIRALDTRELGRLFTYDINPSEDNVFKIKQEVKQKNKLSFVHAFALGNYSYIAFNNDANSGLKESQPNSVLARICLDTDAPRRAAESRKLTESYAQMGLRCGTYTRLLSVSPAELRAETFLFAVFGRADGRAAVCVFRVAEVEEMIRQGRRNCSHGPNSDVQVLDSVIQGSGAECEGKGSIMLQLQTDQLNCGAAHLQHPLALRRPLRARPLYEAQGLSSVAVDSAHNHTLMFLGSRGRLHKVSLHSNFSVSQQWSLRLPANEPVHHIMTFDPSDRTYLYVMTSHHLLRVRVSSCEQYSSCGDCLSAGDPHCGWCTLERRCSVQQDCSSVSLSRSWISISEGVQQCPSMTITPAEISRSAEMRDVGILVAGSVPDLQGLRVECDYGMGISTNATVHLDYGTSHIQTCPLPPAHTLPTIPSGTDHVTVPVSINANGVSVVSGRFIIYDCERTGQIHSTTACTSCLSSAWRCFWDPQLHQCVSSKNNTQQLLQNSSSCPSMVAYEVPPLPTGSSPVFSLELKNVEHGTALECVYSDGQQYRAQWLDGPWVNCSGATLKTTQWSESFSLNLRRAGDATYIDNPQRMTVEVYSCSAGVSDCSQCLGRAALGQECVWCEHICRQQDQCPTTSSARCPAPLIHKIEPLSGPLTGGTLLTVTGKNLGHRADQLSVSIGGVTCHTLPQQYTVSIRLVCETDASLEQMSGQVTVGVAGDAVGVSKEQFSFVEPRLLRFSPAQGPLAGGTTLTIQGQFLDAGSTTDIQINNTHTCSIRTRSSEIIRCVMPAAASAENVSVCVVFDGRPCVSASPNFTFSYQKNPTISHIWPSRSYISGGRSISVSGVGFDLVQSVVMEVPGVGQTNCSCVSSTLILCQSPAADQAQQTAVLFSLNGVQYRDAASAGLEEGEQPHTQRYSFQYVEDPQFYTANKEKLIKHHPGEPLILIINKGPSDLDLTLDEYSVTIGSDLCDITFHNQQLFHCSINRSLSASTGELPVTVRVGHFQKVIAMVQMGGGSELAIVVSIVVCCVLLLLCTVALVVYCTKSRRAERYWQKTLVQMEEMESQIRDEIRKGFAELQTDMTDLTKELSRTQGIPFLEYKQFVTRTFFPKMCCDYERRLVQPVYENDPLGPRAHSETHPLLQDWQPAGSVRPNLEEGITLFSTLLNNKHFLVTFVHALEQQRDFAVRDRCNLASLLTIALHGKLEYYTSIMKDLLVDLIDASATKNPKLMLRRTESVVEKMLTNWMSICMYSYLKETVGEPFFLLLCAIKQQINKGSIDVLTGKARYTLNEEWLLRENIEAKPQNVMVSFQGLGMDSVCVRVMNSDTICQVKEKILEAFYKNLPYSQWPREEDVELEWFPEGRSSRILQDLDDSSVMEDGRKKLNTVFHYQIPDGASLAMSLKDKRENTLGRVKDLDTEKYVHLVLPHDELMESRKTHRQSQRKKVLPEIYLTRLLSTKGTLQKFLDDLFQAILSVPAENPPLAIKYFFDFLEEQADKRGITDPDTLHIWKTNSLPLRFWVNILKNPQFVFDIEKTDHMDACLSVIAQAFIDACSLSDLQLGKDSPTNKLLYAKEIPEYKKRVQSFYRQIQELPALSEQEMNTHLAEESRKHRNEFNTTLALTEVYKYAKRYRAQVASALDSNPTARRTQLQHKFEQVIALVEDNIYECSSEA*

**Zebrafish Plxnd1^skt6^** (1906 aa; mutant)

MPPSAADRRSALALLSALLLAALQTRSAGALHVQQAFATPGRTNNFALDAASGRVYLAAVNNLYQLNATLALEVEMRTGPVLDNPLCHAPQLPQATCEHQKTLTDNHNKLLALDRAQDVLLACGSVYQGFCELRRLENVSRLAVQFPQDGATVFPSMLNIAANHENASTVGLVFRTHGGSPRLLVGATYTGMGTEYFPKNHSKEDLRFENTPEIAIRALDTRELGRLFTYDINPSEDNVFKIKQEVKQKNKLSFVHAFALGNYSYIAFNNDANSGLKESQPNSVLARICLDTDAPRRAAESRKLTESYAQMGLRCGTYTRLLSVSPAELRAETFLFAVFGRADGRAAVCVFRVAEVEEMIRQGRRNCSHGPNSDVQVLDSVIQGSGAECEGKGSIMLQLQTDQLNCGAAHLQHPLALRRPLRARPLYEAQGLSSVAVDSAHNHTLMFLGSRGRLHKVSLHSNFSVSQQWSLRLPANEPVHHIMTFDPSDRTYLYVMTSHHLLRVRVSSCEQYSSCGDCLSAGDPHCGWCTLERRCSVQQDCSSVSLSRSWISISEGVQQCPSMTITPAEISRSAEMRDVGILVAGSVPDLQGLRVECDYGMGISTNATVHLDYGTSHIQTCPLPPAHTLPTIPSGTDHVTVPVSINANGVSVVSGRFIIYDCERTGQIHSTTACTSCLSSAWRCFWDPQLHQCVSSKNNTQQLLQNSSSCPSMVAYEVPPLPTGSSPVFSLELKNVEHGTALECVYSDGQQYRAQWLDGPWVNCSGATLKTTQWSESFSLNLRRAGDATYIDNPQRMTVEVYSCSAGVSDCSQCLGRAALGQECVWCEHICRQQDQCPTTSSARCPAPLIHKIEPLSGPLTGGTLLTVTGKNLGHRADQLSVSIGGVTCHTLPQQYTVSIRLVCETDASLEQMSGQVTVGVAGDAVGVSKEQFSFVEPRLLRFSPAQGPLAGGTTLTIQGQFLDAGSTTDIQINNTHTCSIRTRSSEIIRCVMPAAASAENVSVCVVFDGRPCVSASPNFTFSYQKNPTISHIWPSRSYISGGRSISVSGVGFDLVQSVVMEVPGVGQTNCSCVSSTLILCQSPAADQAQQTAVLFSLNGVQYRDAASAGLEEGEQPHTQRYSFQYVEDPQFYTANKEKLIKHHPGEPLILIINKGPSDLDLTLDEYSVTIGSDLCDITFHNQQLFHCSINRSLSASTGELPVTVRVGHFQKVIAMVQMGGGSELAIVVSIVVCCVLLLLCTVALVVYCTKSRRAERYWQKTLVQMEEMESQIRDEIRKGFAELQTDMTDLTKELSRTQGIPFLEYKQFVTRTFFPKMCCDYERRLVQPVYENDPLGPRAHSETHPLLQDWQPAGSVRPNLEEGITLFSTLLNNKHFLVTFVHALEQQRDFAVRDRCNLASLLTIALHGKLEYYTSIMKDLLVDLIDASATKNPKLMLRRTESVVEKMLTNWMSICMYSYLKETVGEPFFLLLCAIKQQINKGSIDVLTGKARYTLNEEWLLRENIEAKPQNVMVSFQGLGMDSVCVRVMNSDTICQVKEKILEAFYKNLPYSQWPREEDVELEWFPEGRSSRILQDLDDSSVMEDGRKKLNTVFHYQIPDGASLAMSLKDKRENTLGRVKDLDTEKYVHLVLPHDELMESRKTHRQSQRKKVLPEIYLTRLLSTKGTLQKFLDDLFQAILSVPAENPPLAIKYFFDFLEEQADKRGITDPDTLHIWKTNSLPLRFWVNILKNPQFVFDIEKTDHMDACLSVIAQAFIDACSLSDLQLGKDSPTNKLLYAKEIPEYKKRVQSFYRQIQELPALSEQEMNTHLAEESRKHRNEFNTTLALTEVYKYAKRYRAQVASALDSNPTARRTQLQHKFEQVIALVEDNIYESARPEHTHTHTHTHTPECLETACLKGQFPLK*

**C. Human PLXND1 proteins encoded by the WT and mutant alleles in HUVEC/TERT2 cells**. Residues in the mutant protein that differ from the WT protein are indicated in red. Related to **Figure 7-figure supplement 2**.

**Human PLXND1** (1925 aa; wild-type; NCBI Reference Sequence: NP_055918.2)

MAPRAAGGAPLSARAAAASPPPFQTPPRCPVPLLLLLLLGAARAGALEIQRRFPSPTPTNNFALDGAAGTVYLAAVNRLYQLSGANLSLEAEAAVGPVPDSPLCHAPQLPQASCEHPRRLTDNYNKILQLDPGQGLVVVCGSIYQGFCQLRRRGNISAVAVRFPPAAPPAEPVTVFPSMLNVAANHPNASTVGLVLPPAAGAGGSRLLVGATYTGYGSSFFPRNRSLEDHRFENTPEIAIRSLDTRGDLAKLFTFDLNPSDDNILKIKQGAKEQHKLGFVSAFLHPSDPPPGAQSYAYLALNSEARAGDKESQARSLLARICLPHGAGGDAKKLTESYIQLGLQCAGGAGRGDLYSRLVSVFPARERLFAVFERPQGSPAARAAPAALCAFRFADVRAAIRAARTACFVEPAPDVVAVLDSVVQGTGPACERKLNIQLQPEQLDCGAAHLQHPLSILQPLKATPVFRAPGLTSVAVASVNNYTAVFLGTVNGRLLKINLNESMQVVSRRVVTVAYGEPVHHVMQFDPADSGYLYLMTSHQMARVKVAACNVHSTCGDCVGAADAYCGWCALETRCTLQQDCTNSSQQHFWTSASEGPSRCPAMTVLPSEIDVRQEYPGMILQISGSLPSLSGMEMACDYGNNIRTVARVPGPAFGHQIAYCNLLPRDQFPPFPPNQDHVTVEMSVRVNGRNIVKANFTIYDCSRTAQVYPHTACTSCLSAQWPCFWCSQQHSCVSNQSRCEASPNPTSPQDCPRTLLSPLAPVPTGGSQNILVPLANTAFFQGAALECSFGLEEIFEAVWVNESVVRCDQVVLHTTRKSQVFPLSLQLKGRPARFLDSPEPMTVMVYNCAMGSPDCSQCLGREDLGHLCMWSDGCRLRGPLQPMAGTCPAPEIRAIEPLSGPLDGGTLLTIRGRNLGRRLSDVAHGVWIGGVACEPLPDRYTVSEEIVCVTGPAPGPLSGVVTVNASKEGKSRDRFSYVLPLVHSLEPTMGPKAGGTRITIHGNDLHVGSELQVLVNDTDPCTELMRTDTSIACTMPEGALPAPVPVCVRFERRGCVHGNLTFWYMQNPVITAISPRRSPVSGGRTITVAGERFHMVQNVSMAVHHIGREPTLCKVLNSTLITCPSPGALSNASAPVDFFINGRAYADEVAVAEELLDPEEAQRGSRFRLDYLPNPQFSTAKREKWIKHHPGEPLTLVIHKEQDSLGLQSHEYRVKIGQVSCDIQIVSDRIIHCSVNESLGAAVGQLPITIQVGNFNQTIATLQLGGSETAIIVSIVICSVLLLLSVVALFVFCTKSRRAERYWQKTLLQMEEMESQIREEIRKGFAELQTDMTDLTKELNRSQGIPFLEYKHFVTRTFFPKCSSLYEERYVLPSQTLNSQGSSQAQETHPLLGEWKIPESCRPNMEEGISLFSSLLNNKHFLIVFVHALEQQKDFAVRDRCSLASLLTIALHGKLEYYTSIMKELLVDLIDASAAKNPKLMLRRTESVVEKMLTNWMSICMYSCLRETVGEPFFLLLCAIKQQINKGSIDAITGKARYTLNEEWLLRENIEAKPRNLNVSFQGCGMDSLSVRAMDTDTLTQVKEKILEAFCKNVPYSQWPRAEDVDLEWFASSTQSYILRDLDDTSVVEDGRKKLNTLAHYKIPEGASLAMSLIDKKDNTLGRVKDLDTEKYFHLVLPTDELAEPKKSHRQSHRKKVLPEIYLTRLLSTKGTLQKFLDDLFKAILSIREDKPPLAVKYFFDFLEEQAEKRGISDPDTLHIWKTNSLPLRFWVNILKNPQFVFDIDKTDHIDACLSVIAQAFIDACSISDLQLGKDSPTNKLLYAKEIPEYRKIVQRYYKQIQDMTPLSEQEMNAHLAEESRKYQNEFNTNVAMAEIYKYAKRYRPQIMAALEANPTARRTQLQHKFEQVVALMEDNIYECYSEA*

**Human PLXND1 mutant allele-1 in *PLXND1* gRNA KO#1** (***PLXND1^1KO#1^***; 178 aa)

MAPRAAGGAPLSARAAAASPPPFQTPPRCPVPLLLLLLLGAARAGALEIQRRFPSPTPTNNFALDGAAGTVYLAAVNRLYQLSGANLSLEAEAAVGPVPDSPLCHAPQLPQASCEHPRRLTDNYNKILQLDPGQGRSCAGPSTRASASCGAGATSRPWPCASRPPRRPPSPSRCSPAC*

**Human PLXND1 mutant allele-2 in *PLXND1* gRNA KO#1** (***PLXND1^2KO#1^***; 179 aa)

MAPRAAGGAPLSARAAAASPPPFQTPPRCPVPLLLLLLLGAARAGALEIQRRFPSPTPTNNFALDGAAGTVYLAAVNRLYQLSGANLSLEAEAAVGPVPDSPLCHAPQLPQASCEHPRRLTDNYNKILQLDPGQGQLSCAGPSTRASASCGAGATSRPWPCASRPPRRPPSPSRCSPAC*

**Human PLXND1 mutant allele-1 in *PLXND1* gRNA KO#2** (***PLXND1^1KO#2^***; 523 aa)

MAPRAAGGAPLSARAAAASPPPFQTPPRCPVPLLLLLLLGAARAGALEIQRRFPSPTPTNNFALDGAAGTVYLAAVNRLYQLSGANLSLEAEAAVGPVPDSPLCHAPQLPQASCEHPRRLTDNYNKILQLDPGQGLVVVCGSIYQGFCQLRRRGNISAVAVRFPPAAPPAEPVTVFPSMLNVAANHPERVHRGASSASRRGRGGQPPARGRHVHRLRQLLLPAQPQPGGPPLREHARDRHPLPGHARRPGQALHLRPQPLRRQHPQDQAGRQGAAQAGLRERLPAPVRPAAGCTVLRVPGAQQRGARGRQGEPGAEPAGAHLPAPRRRRRQEAHRVLHPVGLAVRGRRGPRRPLQPPGVGLPSPGAALCCLRAAPGVPRGPRCSGRTLRLPLRRRASRHSCAHRLLRGTGARRGGGARQRGAGHGTGLRAQAQHPAPARAAGLWSCSPAAPAVHPAAPEGHARVPRPGPHLRGRGQRQQLHSGLPGHGQREASQDQPEREHAGGEQAGGDCGLWGARAPCHAV*

**Human PLXND1 mutant allele-2 in *PLXND1* gRNA KO#2** (***PLXND1^2KO#2^***; 519 aa)

MAPRAAGGAPLSARAAAASPPPFQTPPRCPVPLLLLLLLGAARAGALEIQRRFPSPTPTNNFALDGAAGTVYLAAVNRLYQLSGANLSLEAEAAVGPVPDSPLCHAPQLPQASCEHPRRLTDNYNKILQLDPGQGLVVVCGSIYQGFCQLRRRGNISAVAVRFPPAAPPAEPVTVFPSMLNVERVHRGASSASRRGRGGQPARGRHVHRLRQLLLPAQPQPGGPPLREHARDRHPLPGHARRPGQALHLRPQPLRRQHPQDQAGRQGAAQAGLRERLPAPVRPAAGCTVLRVPGAQQRGARGRQGEPGAEPAGAHLPAPRRRRRRQEAHRVLHPVGLAVRGRRGPRRPLQPPGVGLPSPGAALCCLRAAPGVPRGPRCSGRTLRLPLRRRASRHPSCAHRLLRGTGARRGGGARQRGAGHGTGLRAQAQHPAPARAAGLWSCSPAAPAVHPAAPEGHARVPRPGPHLRGRGQRQQLHSGLPGHGQREASQDQPEREHAGGEQAGGDCGLWGARAPCHAV*
